# Deep Surveying of the Transcriptional and Alternative Splicing Signatures for Decidual CD8^+^ T Cells at the First Trimester of Human Healthy Pregnancy

**DOI:** 10.3389/fimmu.2018.00937

**Published:** 2018-05-04

**Authors:** Weihong Zeng, Xinmei Liu, Zhicui Liu, Ying Zheng, Tiantian Yu, Shaliu Fu, Xiao Li, Jing Zhang, Siming Zhang, Xiaoling Ma, Xiao-Rui Liu, Xiaoli Qin, Asma Khanniche, Yan Zhang, Fuju Tian, Yi Lin

**Affiliations:** ^1^Institute of Embryo-Fetal Original Adult Disease Affiliated to Shanghai Jiao Tong University School of Medicine, The International Peace Maternity and Child Health Hospital, Shanghai Jiao Tong University School of Medicine, Shanghai, China; ^2^Department of Dermatology, Shanghai Tenth People’s Hospital, Tongji University School of Medicine, Shanghai, China; ^3^Out-Patient Operatingroom, The International Peace Maternity and Child Health Hospital, Shanghai Jiao Tong University School of Medicine, Shanghai, China; ^4^School of Life Science, Tongji University, Shanghai, China; ^5^Shanghai Institute of Immunology, Shanghai Jiao Tong University School of Medicine, Shanghai, China; ^6^Department of Obstetrics and Gynecology, Renmin Hospital of Wuhan University, Wuhan, China

**Keywords:** human decidual CD8^+^ T cells, early healthy pregnancy, transcriptome and alternative splicing, high-throughput mRNA sequencing, functional feature

## Abstract

Decidual CD8^+^ (dCD8) T cells have been proposed to play important roles in immune protection against the invading pathogens and in tolerance toward the growing semi-allogeneic fetus during early pregnancy. However, their phenotypic and functional characteristics remain poorly defined. Here, we performed the first analysis of the transcriptional and alternative splicing (AS) signatures for human first-trimester dCD8 T cells using high-throughput mRNA sequencing. Our data revealed that dCD8 T cells have distinct transcriptional and AS landscapes when compared with their autologous peripheral blood CD8^+^ (pCD8) T counterparts. Furthermore, human dCD8 T cells were observed to contain CD8-Treg and effector-memory T-cell subsets, and display enhanced functionality in terms of degranulation and cytokine production on a per-cell basis. Additionally, we have identified the novel splice junctions that use a high ratio of the non-canonical splicing motif GC-AG and found that AS is not a major contributor to the gene expression-level changes between paired pCD8 and dCD8 T cells. Together, our findings not only provide a comprehensive framework of the transcriptional and AS landscapes but also reveal the functional feature of human dCD8 T cells, which are of great importance in understanding the biology of these cells and the physiology of human healthy pregnancy.

## Introduction

How the semi-allogeneic fetus can survive and grow within the maternal body but without immune rejection has been a long-standing puzzle for reproductive biologists and immunologists (1). During a successful pregnancy in placental mammals, the mucosal surface of the uterus (the endometrium) goes through decidualization upon implantation to form the decidua, a specialized uterine stromal tissue surrounding the implanted conceptus (2). The immune cells that populate the decidua are believed to make an important contribution to the maintenance of successful pregnancies, and an improved understanding of the composition, phenotype and function of these cells will provide novel insights into the physiology of normal pregnancy, and the pathogenesis of pregnancy-related complications and poor postnatal health (1–3).

At the first trimester of human healthy pregnancy, T cells constitute approximately 5–20% of total leukocytes in the decidua where CD8^+^ T cells are the most abundant T-cell subset (2, 4, 5). Previous studies have shown that decidual CD8^+^ (dCD8) T cells play important roles in promoting embryo implantation and trophoblast invasion, maintaining maternal tolerance toward the growing semi-allogeneic fetus, as well as in providing immune protections for both mother and fetus against the invading pathogens, during a successful early pregnancy (6–8). However, the molecular and functional features of these cells during early pregnancy remain largely unknown (4, 5, 8). In comparison with peripheral blood CD8^+^ (pCD8) T cells, dCD8 T cells during early pregnancy were observed to express decreased protein levels of perforin and granzyme B (4, 9) but increased levels of programmed cell death-1 (PD-1) and T-cell immunoglobulin mucin-3, indicating that dCD8 T cells lack effector functions and complete activation that contribute to tolerating the fetal allo-antigens (10, 11). Whereas, other studies showed that early dCD8 T cells upregulate expression of activation antigens such as CD69, HLA-DR, CD38, interleukin-2 receptor-alpha (IL-2Rα, also known as CD25) and IL-2Rβ (CD122) (12) as well as inducible costimulator (ICOS) (13), and exhibit cytolytic ability against P815 and K562 target cells (7), suggesting that dCD8 T cells are regionally activated and display a cytolytic activity which act to regulate the invasion of extravillous trophoblast cells during early pregnancy. A deep understanding of the phenotype and functionality of human dCD8 T cells will help in better characterization of their roles during healthy pregnancies.

As a recently developed and revolutionary approach to uncover the complexity of mammalian transcriptomes, high-throughput mRNA sequencing (mRNA-Seq) allows analysis of both gene expression and alternative splicing (AS) levels with a high reliability and sensitivity *via* mRNA transcript abundance (14, 15). As an ubiquitous and crucial mechanism to regulate gene expression in mammals, AS plays important roles in physiology and disease, and is proposed as a principal driver of the evolution of phenotypic and functional complexity (16–18). It has also been demonstrated that AS is an important factor in shaping T-cell biology and effector function. Moreover, numerous immune-responsive genes tend to undergo AS, which acts on multiple layers ranging from the cell-surface receptors/adapter proteins, cytokines/chemokines, and intracellular signaling proteins to transcription factors (16, 17). However, the AS complexity of dCD8 T cells during early healthy pregnancy has never been elucidated.

Herein, we aimed not only to investigate the transcriptional and AS signatures but also to determine the functional feature of paired pCD8 and dCD8 T cells at the first trimester of human healthy pregnancy by using high-throughput mRNA-Seq and flow cytometry, respectively.

## Materials and Methods

### Human Subjects and Study Approval

Twenty-seven healthy women at the first trimester of pregnancy were recruited for this study. All of them had never undergone preterm labor, spontaneous abortion nor preeclampsia in any pregnancy. At the time of specimen collection, they were undergoing early elective surgical abortion at the Department of Obstetrics and Gynecology in the International Peace Maternity and Child Health Hospital of China Welfare Institute (Shanghai, China). Maternal peripheral blood samples were harvested from the median cubital vein before pregnancy termination and then collected immediately in EDTA-anticoagulant tubes (BD, USA). Autologous decidual tissues were collected by uterine aspiration and curettage, and were stored in sterile ice-cold phosphate-buffered saline (PBS). Samples from three women (mean age 26 years, range 22–28 years; mean gestational day 50, range 44–58 days) were used for high-throughput mRNA-Seq, and five others (mean age 30 years, range 22–39; mean gestational day 45, range 38–50) were enrolled to validate the mRNA-Seq data and evaluate CD8-Treg frequency. Meanwhile, samples from another four women (mean age 34 years, range 30–39; mean gestational day 45, range 43–50 days) were used to determine the IFN-γ and IL-17A secretion and memory phenotype, and five others (mean age 25 years, range 19–33; mean gestational day 58, range 44–75) were applied to evaluate CD107a expression in CD8^+^ T cells (Figure S1 in Supplementary Material). Statistical analyses revealed that the differences in both age and gestational day are not statistically significant across these four cohorts (Figure S2 in Supplementary Material). The study was approved by the Medical Ethics Committee of the International Peace Maternity and Child Health Hospital of China Welfare Institute and all experiments were performed according to the principles of the Declaration of Helsinki. Informed consent was assigned individually from all participants before enrollment.

### Isolation of Decidual and Peripheral Blood Mononuclear Cells (PBMCs)

We isolated the decidual mononuclear cells (DMCs) using the procedure of non-enzymatic leukocytes separation, as mentioned in previous studies (12, 18–22). Vacuum-aspirated abortion tissues were washed in sterile ice-cold PBS; and the decidual tissue that was separated macroscopically from chorionic villus was cut into small pieces (<1 mm^3^) using ocular scissors (10 cm) and filtered through a 74-µm nylon mesh filter to obtain DMCs. Both PBMCs and DMCs were separated by density gradient centrifugation by Lymphoprep™ (AS1114546, Axis-shield) according to the manufacturer’s recommendation.

### Isolation of CD8^+^ T Cells

Human DMCs and PBMCs were incubated with fluorescein-conjugated anti-human monoclonal antibodies (mAbs) including anti-CD3 FITC (clone: UCHT1; BD Biosciences, USA), anti-CD4 V500 (RPA-T4; BD Biosciences, USA), and anti-CD8a PerCP/Cy5.5 (HIT8a; BioLegend, USA) in 1 mL PBS containing 3% (v/v) fetal bovine serum (FBS) at 4°C for 30 min. Paired dCD8 and pCD8 T cells were isolated from the DMCs and PBMCs, respectively, by sorting on an FACSAria III (BD Biosciences, USA) based on the surface expression of CD3, CD4, and CD8 (CD3^+^CD8^+^CD4^−^) with a purity always greater than 95%.

### RNA Preparation

Total RNA was extracted from the isolated CD8^+^ T cells using Trizol^®^ Reagent (Invitrogen, USA) and was purified using RNeasy^®^ Micro Kit (Qiagen, Germany) according the manufacturers’ protocols. RNA degradation was checked on 1% agarose gels. The RNA purity, concentration, and integrity were evaluated by the NanoPhotometer^®^ spectrophotometer (IMPLEN, USA), the Qubit^®^ RNA Assay Kit in Qubit^®^ 2.0 Flurometer (Life Technologies, USA), and the RNA Nano 6000 Assay Kit of Agilent Bioanalyzer 2100 system (Agilent Technologies, USA), respectively, as previous studies performed (18, 23).

### Library Preparation, Clustering, and mRNA-Seq

We carried out the library preparation, clustering, and mRNA-Seq as described in previous studies (18, 23). An amount of 200 ng RNA per sample was utilized as input material for the RNA sample preparation. cDNA libraries were constructed by using the NEBNext^®^ Ultra™ RNA Library Prep Kit for Illumina^®^ (NEB, USA) according to the manufacturer’s instruction and index codes were added to attribute sequences to each sample. The products were purified through the AMPure XP system (Beckman Coulter Genomics, UK) and library quality was evaluated on the Agilent Bioanalyzer 2100 system (Agilent Technologies, USA).

Clustering of the index-coded libraries was achieved through a HiSeq 2500 PE Cluster Kit of cBot Cluster Generation System (Illumia, USA), following the manufacturer’s recommendations. After cluster generation, the prepared libraries were sequenced on an Illumina Hiseq 2500 platform and 125 bp paired-end reads were produced. The complete data are deposited in NCBI Gene Expression Omnibus with accession number GSE105064.

### Sequence Alignment and Splice Junction Analysis

Sequenced reads were aligned to the human reference genome (hg19 version) using the STAR software package (18, 24), and splice junctions were identified. A splice junction was defined as “unannotated” if not present in the University of California Santa Cruz (UCSC) expressed sequence tag (EST)/mRNA data set (24). These “unannotated” splice junctions were further compared with the GENCODE data set (25) to identify the novel splice junctions. To analyze the splicing motif on the splice junctions, a 5-bp sequence around each splice site (3 bp to the exon and 2 bp to the intron) was considered (24), and the proportion of each splice site motifs GT-AG, GC-AG, AT-AC, and others was evaluated by the number of junctional read counts. Sequence logos of splice site motifs were generated by WebLogo.

### Gene Expression and AS Analyses

Both gene expression and AS analyses were performed as in our previous study (18). For gene expression analysis, the number of reads falling in the meta-gene, which was created by merging exons from all isoforms of a gene, was counted using HTSeq-count (18). To identify the differentially expressed genes (DEGs), the DESeq2 algorithm was performed, and the genes with a *P* < 0.05 (paired test) were considered to be significantly differentially expressed and the adjusted *P*-values for controlling false discovery rate (FDR) were also calculated (2, 18) (Table S1 in Supplementary Material).

For identification and differential expression analysis of AS events, rMATS (v3.2.1 beta) paired model was applied (21), using the splice junction counts as the input in our mRNA-Seq dataset. The hg19 genome was used as reference genome, and transcript annotation from the NCBI Reference Sequence (26) was employed for AS events annotation. We determined the “exon inclusion level” (Ψ) value by the percentage of the density of exon inclusion transcripts among the density of exon inclusion transcripts plus exon skipping transcripts, as previously described (18, 21). Differential AS events were those with a |ΔΨ| > 0.05 and an FDR < 0.05, including two sets: upregulated AS set (those with an FDR < 0.05 and a ΔΨ > 0.05) and downregulated AS set (FDR < 0.05 and ΔΨ < −0.05).

### Heatmap, Gene Ontology Annotation, KEGG Pathway, and Gene Set Enrichment Analysis

We performed heatmap analysis of differential genes using R, and functional enrichment analyses including GO annotation and KEGG pathway enrichment by using the Database for Annotation, Visualization, and Integrated Discovery (http://david.ncifcrf.gov/home.jsp). *P* < 0.05 were considered as the significance threshold. GSEA was carried out by using the GSEA-P software, MSigDB 1.0, as previously performed (18, 27).

### Flow Cytometry

Fluorescein-conjugated anti-human mAbs including anti-CD3 FITC (clone: UCHT1), anti-CD4 V500 (RPA-T4), anti-CD8 APC/Cy7 (SK1), and anti-CD279 (PD-1) PE (EH12.1, also known as EH12) were purchased from BD Pharmingen; and anti-CD8a PerCP/Cy5.5 (HIT8a), anti-CD38 FITC (HIT2), anti-CD63 FITC (H5C6), anti-CD69 APC (FN50), anti-CD122 (IL-2Rβ) APC (TU27), anti-CD183 (CXCR3) PE/Cy7 (G025H7), anti-CD192 (CCR2) APC/Cy7 (K036C2), anti-CD196 (CCR6) PE (G034E3), anti-CD197 (CCR7) PE (G043H7), anti-CD276 (B7-H3) PE (DCN.70), anti-CD45RO FITC (UCHL1), anti-CD197 (CCR7) PE/Cy7 (G043H7), anti-IFN-γ APC/Cy7 (4S.B3), anti-IL-17A FITC (BL168), and anti-CD107a (LAMP-1) APC/Cy7 (H4A3) were purchased from BioLegend.

We carried out cell-surface and intracellular staining by multicolor flow cytometry, as previously described (18, 19, 28). For cell-surface staining, the isolated mononuclear cells were incubated with different cocktails of anti-human mAbs in 100 µL PBS containing 3% FBS at room temperature for 30 min. For intracellular cytokine and anti-CD107a staining, cells were resuspended in R10 media (RPMI 1640 supplemented with 10% FBS), and stimulated with 81 nM phorbol-12-myristate-13-acetate (PMA) and 1.34 µM ionomycin in the presence of brefeldin (10.6 µM), monensin (2 µM) (eBiosciences, USA), and anti-CD107a (LAMP-1) APC/Cy7 (5 μL/well) at 37°C for 4 h; cells were stained for surface markers (anti-CD3 and anti-CD8) and then for cytokines using anti-IFN-γ and IL-17A mAbs after permeabilization with Cytofix/Cytoperm kit (BD Biosciences, USA) following the manufacturer’s recommendation. The immunostained cells were collected and analyzed on fluorescence-activated cell sorting (FACS) Canto II flow cytometer (BD Biosciences, USA) and data were processed using the FlowJo 7.6.1 software.

Statistical analyses were carried out by using the Graphpad Prism 5 software (29). The normality of the data was evaluated by Kolmogorov–Smirnov test. If the data were normally distributed, statistical analysis was performed using paired Student’s *t*-test; otherwise, the Wilcoxon matched pairs test was performed (3). *P* < 0.05 were considered statistically significant. The details are given in the figure legends.

## Results

### The Numbers of DEGs Are Comparable in Human dCD8 Versus pCD8 T Cells

**Figure 1:**
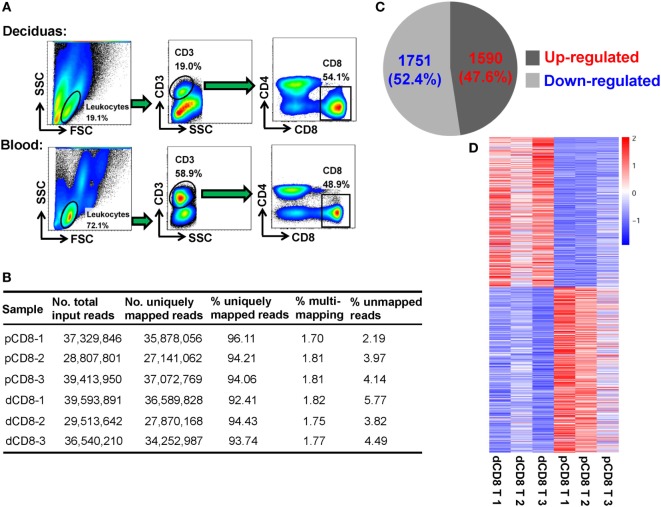
The numbers of DEGs are comparable in human dCD8 versus pCD8 T cells. **(A)** Fluorescence-activated cell sorting was performed to purify the CD8^+^ T cells, based on the phenotype of CD3^+^CD8^+^CD4^−^, in paired decidual and peripheral blood samples from three healthy women at the first trimester of normal pregnancy (mean gestational day 50, range 44–58). **(B)** Summary of mRNA sequencing data of the isolated human pCD8 and autologous dCD8 T cells. 2 × 125-bp paired-end cDNA fragments were acquired after sequencing the polyadenylated [poly(A)+] enriched mRNAs on an Illumina Hiseq 2500 platform, and the sequenced reads was aligned to human reference genome (hg19 version) by STAR software. **(C)** The number and proportion of DEGs that were upregulated or downregulated in human dCD8 versus pCD8 T cells (*P* < 0.05, paired test). **(D)** Heatmap analysis of DEGs using R. Each row represents one gene and each column represents one sample. Different colors denote the expression levels (from red to blue: decreased expression in dCD8 versus pCD8 T cells). Abbreviations: dCD8 T, decidual CD8^+^ T; pCD8 T, peripheral blood CD8^+^ T; No., number; %, percentage; DEGs, differentially expressed genes.

To obtain a global view of the transcriptional and AS signatures of human dCD8 T cells during early normal pregnancy, we performed high-throughput mRNA-Seq for paired dCD8 and pCD8 T cells *via* Illumina sequencing technology. FACS was used to purify the CD8^+^ T cells (CD3^+^CD8^+^CD4^−^) in peripheral blood and autologous decidual samples from three healthy women at the first trimester of pregnancy (Figure 1A; Figure S1A in Supplementary Material). In total, approximately 210 million 2 × 125-bp paired-end cDNA fragments (ranging from 28 to 40 million reads per sample) were acquired after sequencing the polyadenylate-enriched mRNAs of isolated dCD8 and pCD8 T cells on an Illumina Hiseq 2500 platform (Figure 1B). Of the total sequenced fragments, between 92 and 96% of reads per sample, were mapped uniquely on the human reference genome (hg19 version) (Figure 1B); the percentages of multi-loci mapping and unmapped reads per sample were less than 2 and 6%, respectively, both of which were excluded from further analyses (Figure 1B).

Here, gene expression was quantified by the read count, which is linearly related and represents a good approximation to the abundance of the target transcript (14, 18, 30). Unlike our previous finding in CD4^+^ T cells (18), we observed that there were 3,341 DEGs between human dCD8 and pCD8 T cells, with a comparable number of genes being upregulated (1,590 genes) and downregulated (1,751 genes) in dCD8 versus pCD8 T cells (Figures 1C,D; Table S1 in Supplementary Material).

### Human dCD8 T Cells Upregulate the Genes Involved in M Phase of Mitotic Cell Cycle and Immune System Process but Downregulate Those Related to Metabolic Process

**Figure 2:**
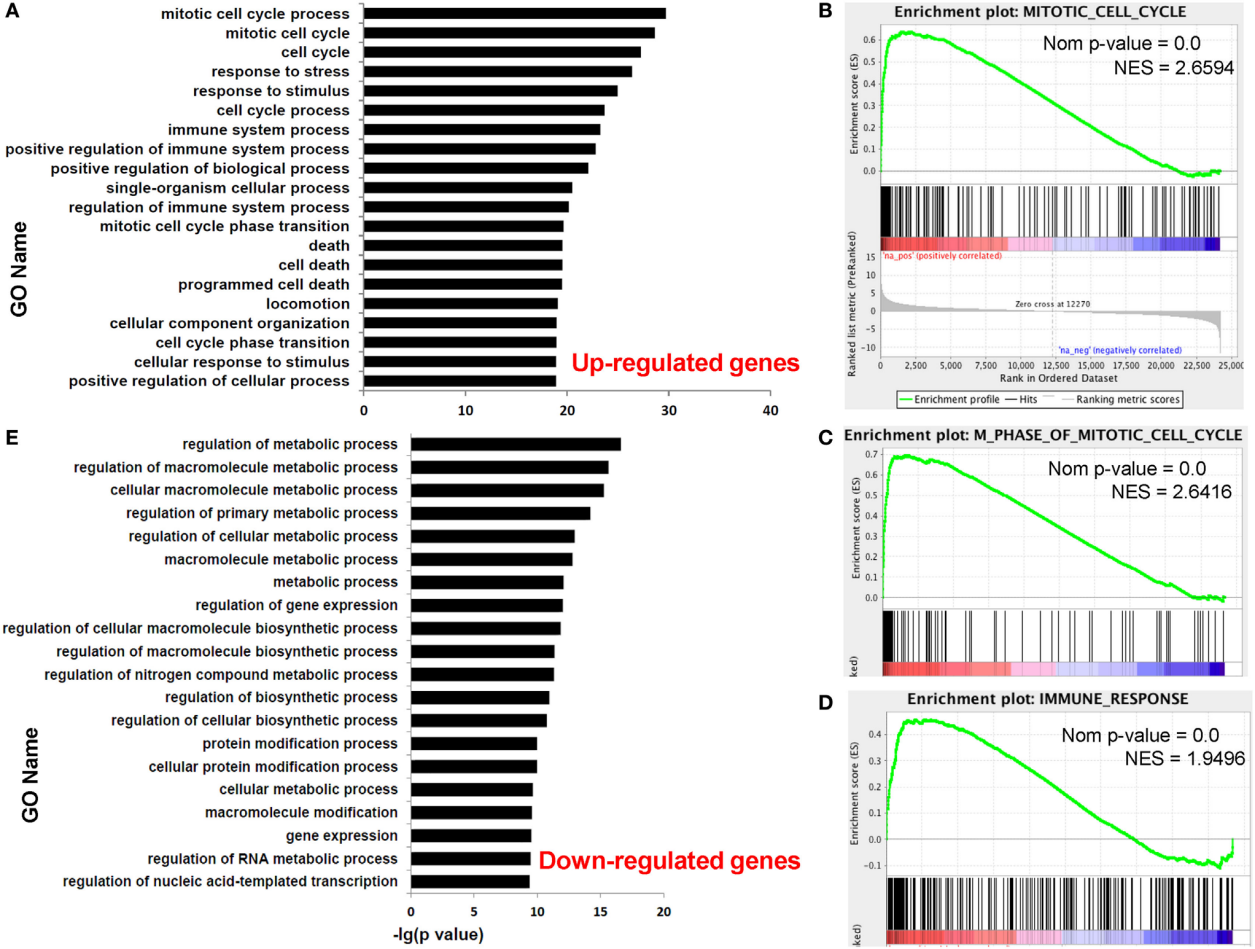
Human dCD8 T cells upregulate the genes involved in M phase of mitotic cell cycle and immune *system* process but downregulate those related to metabolic process. **(A)** The top 20 GO terms (biological process) enriched for the upregulated genes in human dCD8 versus pCD8 T cells. **(B–D)** GSEA plots of gene sets in GO categories including mitotic cell cycle **(B)**, M phase of mitotic cell cycle **(C)**, and immune response **(D)** in human dCD8 versus pCD8 T cells. **(E)** The top 20 GO terms enriched for the downregulated genes in human dCD8 T cells. The NES reflects the degree to which a gene set is upregulated (positive NES) in human dCD8 T cells and the corresponding nom *P*-value is indicated. Abbreviations: GO, gene ontology; dCD8 T, decidual CD8^+^ T; pCD8 T, peripheral blood CD8^+^ T; GSEA, gene set enrichment analysis; Nom, nominal; NES, normalized enrichment score.

Functional enrichment analysis of DEGs between paired dCD8 and pCD8 T cells could provide insights into the molecular and functional features of these cells during early pregnancy. Thus, we performed GO annotation, KEGG pathway, and GSEA analyses for DEGs. GO annotation revealed that the upregulated genes in human dCD8 T cells were most significantly enriched in the terms related to mitotic cell cycle process and immune system process (Figure 2A; Table S2 in Supplementary Material). GSEA analysis also revealed that the gene set of mitotic cell cycle was most significantly and positively enriched in human dCD8 versus pCD8 T cells, followed by M phase of mitotic cell cycle, cell cycle phase, M phase, chromosome segregation, immune response, immune system process, regulation of immune system process, and immune effector process (Figures 2B–D; Figure S3 in Supplementary Material). Furthermore, KEGG pathway analysis showed that the upregulated genes in dCD8 T cells were remarkably enriched in the terms related to cell cycle, antigen processing and presentation, allograft rejection, p53 signaling pathway, and cytokine–cytokine receptor interaction, which was confirmed by GSEA analysis (Figure S4 in Supplementary Material). Thus, these data indicated that human dCD8 T cells upregulate the genes involved in M phase of mitotic cell cycle and immune system process.

Nevertheless, GO annotation revealed that the downregulated genes in human dCD8 T cells were dramatically enriched in the terms involved in metabolic process (Figure 2E; Table S3 in Supplementary Material). In addition, GSEA analysis showed that the gene set of nuclear export was most negatively enriched in dCD8 versus pCD8 T cells, followed by protein processing, protein amino acid autophosphorylation, chromatin remodeling, and establishment and/or maintenance of chromatin architecture (Figure S5 in Supplementary Material). Together, these results indicated that human dCD8 T cells mainly downregulate the genes related to metabolic process.

### Human dCD8 T Cells Show Increased Activation and Proliferation, Display CD8-Treg and Effector-Memory Phenotypes, and Have an Enhanced Functionality

**Figure 3:**
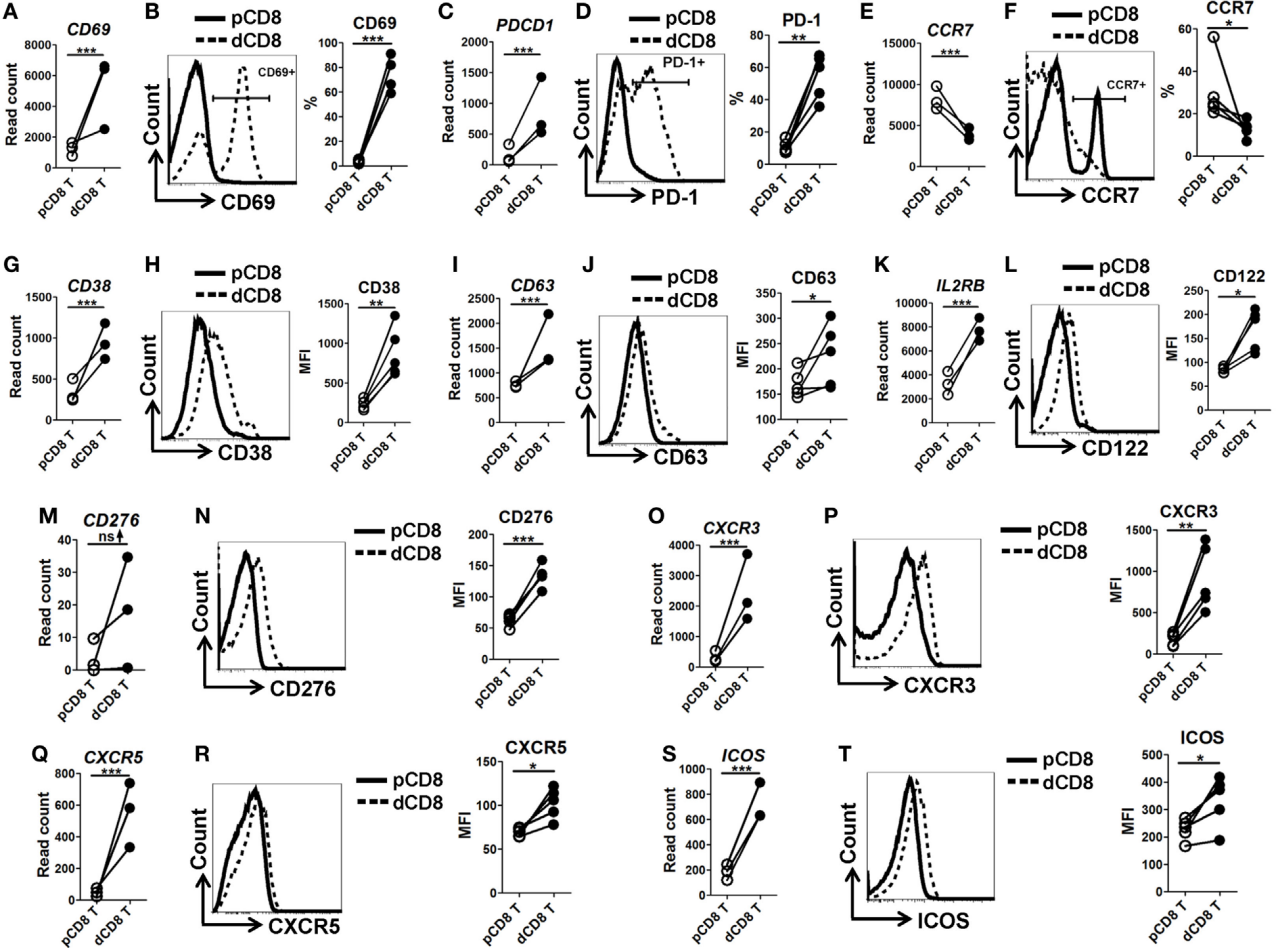
Flow cytometry validation of mRNA-Seq data. **(A,C,E,G,I,K,M,O,Q,S)** Comparison of the gene expression (measured as the read count) of *CD69* **(A)**, *PDCD1* [encoding PD-1, **(C)**], *CCR7* **(E)**, *CD38* **(G)**, *CD63* **(I)**, *IL2RB* [encoding CD122, **(K)**], *CD276* **(M)**, *CXCR3* **(O)**, *CXCR5* **(Q)**, and *ICOS* **(S)** between paired pCD8 and dCD8 T cells from mRNA-Seq data. Differential expression analysis was performed using DESeq2 algorithm (paired test). Each symbol reflects a sample and each line reflects samples from the same person (*n* = 3 per group). **(B,D,F,H,J,L,N,P,R,T)** Representative flow cytometric histograms (left) and cumulative data (right) illustrating the comparison of molecular expression, measured as the percentage and/or geometric MFI, of indicated proteins between paired pCD8 and dCD8 T cells (*n* = 5 per group). The cells are gated in CD8^+^ T cells and the data are representative of two independent experiments. The flow cytometric data were assessed statistically using the paired Student’s *t*-test **(B,D,H,J,L,N,P,R,T)** or Wilcoxon matched pairs test **(F)**. Abbreviations: pCD8 T, peripheral blood CD8^+^ T; dCD8 T, decidual CD8^+^ T; %, percentage; MFI, mean fluorescent intensity; mRNA sequencing; mRNA-Seq.

**Figure 4:**
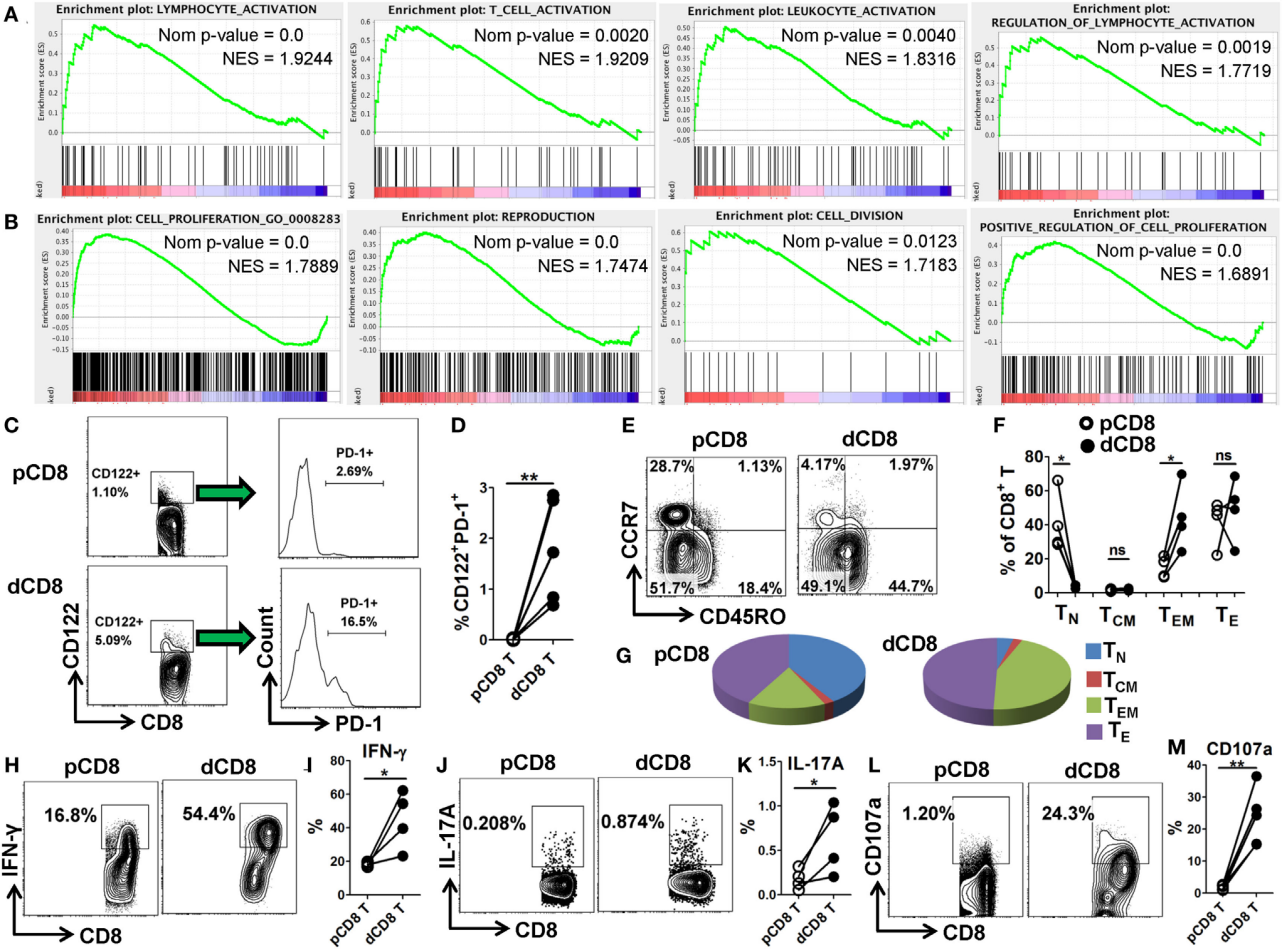
Human dCD8 T cells show increased activation and proliferation, display CD8-Treg and effector-memory phenotypes, and have an enhanced functionality. **(A,B)** GSEA plots of gene sets in GO categories including lymphocyte/T cell/leukocyte activation, regulation of lymphocyte activation **(A)**, and cell proliferation, reproduction, cell division, and positive regulation of cell proliferation **(B)** in human dCD8 versus pCD8 T cells. NES reflects the degree to which a gene set is upregulated (positive NES) in dCD8 cells and the corresponding nom *P*-value is indicated. **(C,D)** Representative flow cytometric plots/histograms **(C)** and cumulative data **(D)** displaying the proportion of CD122^+^PD-1^+^ Treg cells among paired pCD8 and dCD8 T cells (*n* = 5 per group). **(E–G)** Representative flow cytometric plots **(E)**, column chart **(F)**, and pie graphs **(G)** illustrating the proportions of native (T_N_, CD45RO^−^CCR7^+^), effector (T_E_, CD45RO^−^CCR7^−^), central memory (T_CM_, CD45RO^+^CCR7^+^), and effector-memory (T_EM_, CD45RO^+^CCR7^−^) T cells in paired pCD8 and dCD8 T cells (*n* = 5 per group). **(H–K)** Comparison of IFN-γ and IL-17A production between in paired pCD8 and dCD8 T cells as determined by intracellular cytokine staining upon stimulation with PMA and ionomycin *in vitro* (*n* = 4 per group). **(L,M)** Degranulation in PMA-ionomycin activated pCD8 and dCD8 T cells (*n* = 5 per group), was measured by flow cytometric staining with anti-CD107a (LAMP-1) APC/Cy7 (5 μL/well) included in the initial culture medium (RPMI 1640 supplemented with 10% fetal bovine serum). The flow cytometric data were assessed statistically using the paired Student’s *t*-test **(D,I,K,M)** or Wilcoxon matched pairs test **(F)**. Each symbol reflects a sample and each line reflects samples from the same person **(D,F,I,K,M)**. The flow data are representative of two independent experiments. Abbreviations: Nom, nominal; NES, normalized enrichment score; GO, gene ontology; GSEA, gene set enrichment analysis; pCD8 T, peripheral blood CD8^+^ T; dCD8 T, decidual CD8^+^ T; %, percentage; PMA, phorbol-12-myristate-13-acetate.

In order to confirm the mRNA-Seq data, we performed flow cytometry staining for 10 immune-responsive molecules in another 5 healthy women at the first trimester of pregnancy (Figure 3; Figures S1 and S6 in Supplementary Material). Our data showed that human dCD8 T cells highly upregulate expression (protein level) of CD69, PD-1 (encoded by *PDCD1*), CD38, CD63, CD122 (encoded by *IL2RB*), CD276, CXCR3, CXCR5, and ICOS, but downregulate CCR7, agreeing with the mRNA-Seq data (Figure 3; Figures S1 and S6 in Supplementary Material). These flow data indicated that human dCD8 T cells have increased activation and proliferation. Indeed, both GO annotation and GSEA analysis also revealed that the upregulated genes in dCD8 T cells were significantly enriched in the terms related to lymphocyte/T cell/leukocyte activation, regulation of lymphocyte activation, cell proliferation, reproduction, cell division, and positive regulation of cell proliferation (Figures 4A,B; Table S2 in Supplementary Material).

CD8^+^CD122^+^ T cells contain an abundant subset of PD-1^+^ cells that are CD8^+^ Treg cells; these Treg cells (CD8^+^CD122^+^PD-1^+^) are capable of suppressing autoimmunity and alloimmunity mainly through their secretion of IL-10, and are largely CD127 (encoded by *IL-7R*α) negative (31, 32). Additionally, both CD103-expressing and HLA-G-expression CD8^+^ T cells have been classified as novel Treg-cell subsets based on their potent suppressive function (20, 33). Herein, we observed that dCD8 T cells contained a higher proportion of CD122^+^PD-1^+^ Treg cells, and upregulated expression of genes *IL-10, ITGAE* (encoding CD103) and *HLA-G* but downregulated *IL7R*α, when compared with autologous pCD8 T cells (Figures 4C,D; Figure S7 in Supplementary Material), indicating that human dCD8 T cells display a CD8-Treg phenotype.

Consistent with a previous study (4), we found that CD8^+^ T cells in early human decidua significantly increased the proportion of effector-memory T (T_EM_, CD45RO^+^CCR7^−^) cells but decreased native (T_N_, CD45RO^−^CCR7^+^) cells when compared with autologous pCD8 T cells, indicating that human dCD8 T cells consist mainly of T_EM_ cells whereas T_N_ cells are almost absent (Figures 4E–G). Furthermore, dCD8 T cells produce more cytokines IFN-γ and IL-17A, and the degranulation antigen CD107a, upon stimulation with PMA and ionomycin *in vitro* (Figures 4H–M), which might be because of the enrichment of T_EM_ but absence of T_N_ in these cells (Figures 4E–G).

Taken together, these data suggested that human dCD8 T cells during early pregnancy are endowed with increased activation and proliferation as well as elevated functionality, and also display CD8-Treg and T_EM_ phenotypes.

### Novel Splice Junctions Use a High Ratio of the Non-Canonical Splicing Motif GC-AG and Are Enriched in the Genes Related to Immune Response

To explore the magnitude and characteristic of CD8^+^ T-cell specific splice junctions in human early decidua and maternal peripheral blood, we analyzed about 210 million sequencing reads obtained across each sample (Figure 1B). A total of 581,925 splice junctions were identified, of which 149,755 junctions were annotated in the UCSC EST/mRNA dataset (Figure S8A in Supplementary Material). To identify the novel splice junctions, the remaining “unannotated” splice junctions were further compared with the GENCODE dataset (25). Aggregately, 98.5% of the “unannotated” events were detected in the GENCODE dataset, which were hereafter called unannotated splice junctions (425,848 junctions belonging to 15,961 genes); and the remaining 6,322 events (1.5%; belonging to 2,137 genes) were specific to our dataset, which were called novel splice junctions (Figure S8A and Table S4 in Supplementary Material).

**Figure 5:**
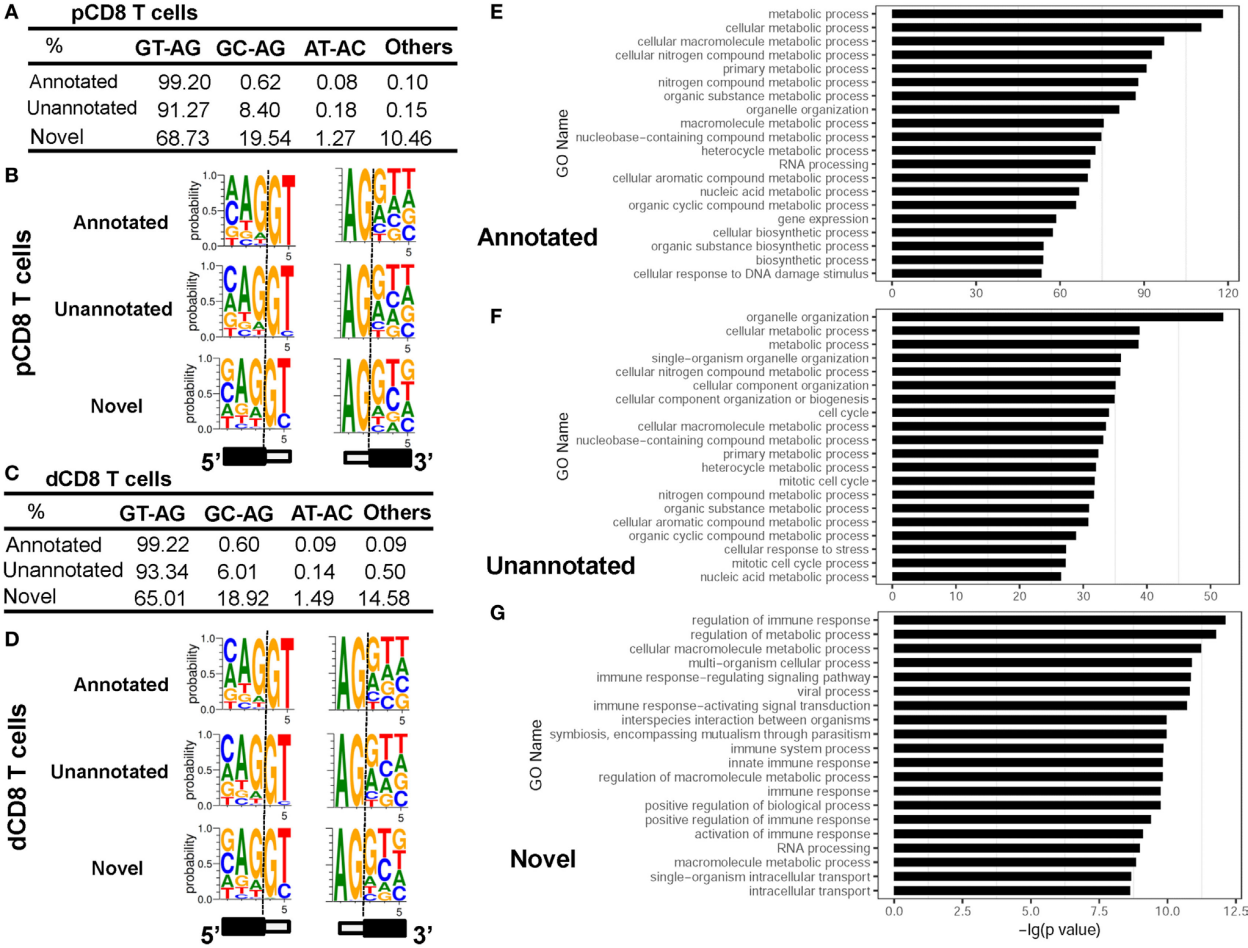
Novel splice junctions use a high ratio of the non-canonical splicing motif GC-AG and are enriched in the genes related to immune response. **(A–D)** Comparison of splice site motifs for annotated, unannotated, and novel splicing junctions in human pCD8 **(A,B)** and dCD8 T cells **(C,D)**. To analyze the splice site motif on each splice junction, a 5-bp sequence around each splice site (3 bp to the exon and 2 bp to the intron) was considered, and the proportion of each splice site motifs GT-AG, GC-AG, AT-AC, and others was evaluated by the number of junctional read counts. Sequence logos of splice site motifs **(B,D)** were generated by WebLogo (black box, exon; white box, intron). **(E–G)** Functional enrichment analyzed the genes containing annotated **(E)**, unannotated **(F)**, or novel **(G)** splice junctions and the top 20 GO terms (biological process) were indicated. Abbreviations: pCD8 T, peripheral blood CD8^+^ T; dCD8 T, decidual CD8^+^ T; %, percentage, GO, gene ontology.

Analyzing the splice sites revealed that the novel splice junctions used a higher ratio of the non-canonical splicing motif GC-AG than the annotated and unannotated splice junctions in both pCD8 and dCD8 T cells (Figures 5A–D; Table S4 in Supplementary Material). Moreover, enrichment analysis showed that the genes containing novel splice junctions were most significantly enriched in the GO terms related to immune system and metabolic processes, whereas both annotated and unannotated splice junctions were mainly enriched in the genes involved in metabolic process (Figures 5E–G). In addition, KEGG pathway analysis also showed that the genes having novel junctions were dramatically enriched in immune response such as antigen processing and presentation, T-cell receptor signaling pathway, allograft rejection, and mTOR signaling pathway (Figure S8B in Supplementary Material).

Overall, we have identified the novel splice junctions specific to human pCD8 and dCD8 T cells, which use a high ratio of the non-canonical splicing motif GC-AG and are enriched in the genes related to immune response.

### Genes in Human dCD8 T Cells Undergo a Comparable Number of Upregulated and Downregulated AS Events

**Figure 6:**
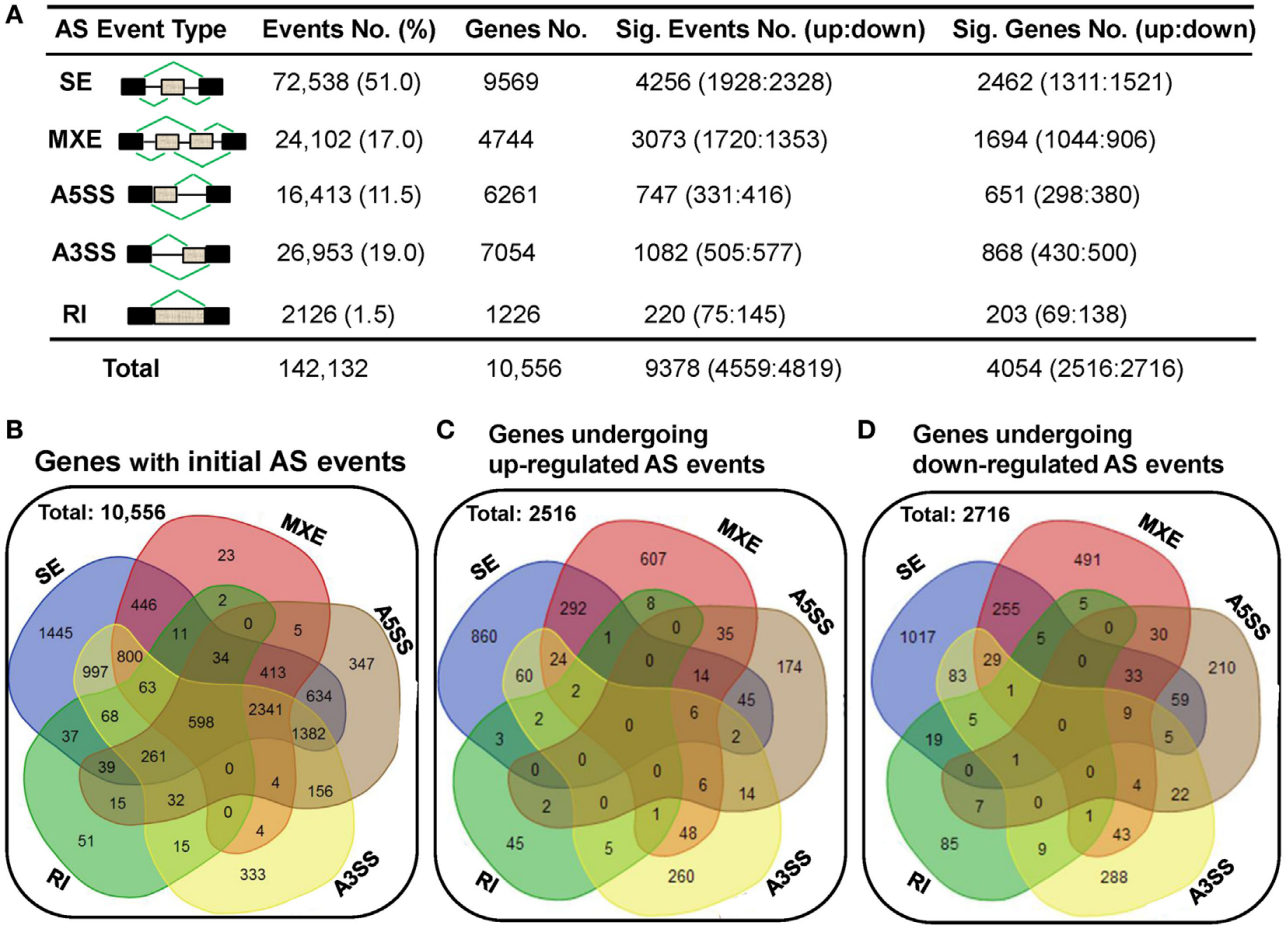
A comparable number of upregulated and downregulated AS events is detected in human dCD8 T cells with respect to autologous pCD8 T cells. **(A)** Summarization of the numbers of initial AS events, statistically significant AS events and of the genes these events belong to. Column 1 illustrates traditional classification of the five most common types of AS events that have been investigated (black box, exon; gray box, intron). Columns 2–5 represent the number of AS events or genes of each type: (2) the initial AS events detected in the combined samples of pCD8 and dCD8 T cells with their frequency among total AS events; (3) genes the initial AS events belong to; (4) statistically significant AS events (FDR < 0.05 with |ΔΨ| > 0.05 between samples) with upregulated (FDR < 0.05 with ΔΨ > 0.05) and downregulated (FDR < 0.05 with ΔΨ < −0.05) events in human dCD8 versus pCD8 T cells; (5) genes showing the differentially expressed AS events. **(B)** Venn diagram of genes showing the five types of initial AS events (including SE, MXE, A5SS, A3SS, and RI) in the combined samples of pCD8 and dCD8 T cells. **(C,D)** Venn diagram of genes displaying upregulated [**(C)**, FDR < 0.05 with ΔΨ > 0.05] or downregulated [**(D)**, FDR < 0.05 with ΔΨ < −0.05] SE, MXE, A5SS, A3SS, and RI events in dCD8 T cells with respect to autologous pCD8 T cells. Abbreviations: AS, alternative splicing; SE, skipped exon; MXE, mutually exclusion exons; A5SS, alternative 5’ splice site; A3SS, alternative 3’ splice site; RI, retained intron; %, percentage; No., number; Sig., significant; up, upregulated; down, downregulated; FDR, false discovery rate.

To elucidate the AS complexity, five basic AS modes including skipped exon (SE), mutually exclusion exons (MXE), alternative 5’ splice site (A5SS), alternative 3’ splice site (A3SS), and retained intron (RI) were investigated (Figure 6A). A total of 142,132 AS events belonging to 10,556 genes were detected in either human dCD8 or pCD8 T cells (Figures 6A,B). There were only 598 genes undergoing all five AS modes, which were enriched in immune response and catabolic process (Figure 6B; Figure S9 in Supplementary Material). Similar to our previous findings in human decidual and circulating CD4^+^ T cells (18), we observed that SE was the primary AS mode, accounting for more than half of all splicing events (51.0%; 9,569 events); whereas RI was the least common (1.5%; 1,226 events) (Figures 6A,B).

Moreover, our data showed that there was a comparable number of upregulated and downregulated AS events in human dCD8 T cells when compared with autologous pCD8 T cells (upregulated events versus downregulated events: 1,928 versus 2,328 of SE, 1,720 versus 1,353 of MXE, 331 versus 416 of A5SS, 505 versus 577 of A3SS, and 4,559 versus 4,819 of total AS events) (Figure 6A; Tables S5–S9 in Supplementary Material). These differentially expressed AS events also belonged to a comparable number of genes (Figures 6A,C,D). In addition, there was a comparable number of genes between those showing one AS mode-specific upregulation and downregulation, as well as between those showing two or more modes-simultaneous upregulation and downregulation, in human dCD8 versus pCD8 T cells (Figures 6C,D).

### Differential AS Events Are Enriched in the Genes Related to Cellular Metabolic Process

**Figure 7:**
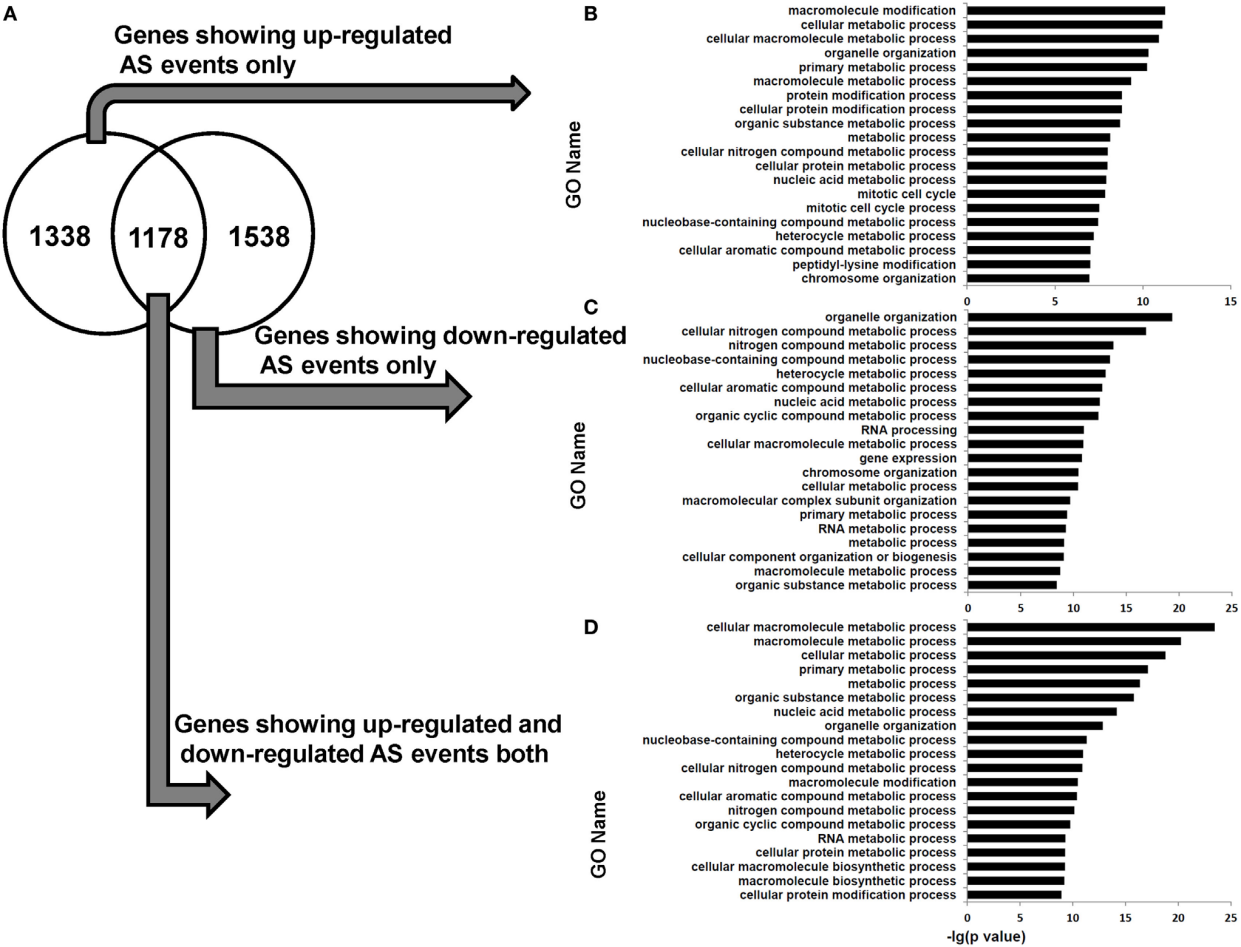
Venn diagram and functional enrichment analysis for the genes showing upregulated and downregulated AS events in human dCD8 T cells. **(A–D)** Venn diagram **(A)** and GO annotation **(B–D)** for the genes showing upregulated (FDR < 0.05 with ΔΨ > 0.05) and downregulated (FDR < 0.05 with ΔΨ < −0.05) AS events in human dCD8 T cells with respect to autologous pCD8 T cells. The five types of AS events including SE, MXE, A5SS, A3SS, and RI were combined together. The top 20 GO terms (biological process) were indicated **(B–D)**. Abbreviations: GO, gene ontology; FDR, false discovery rate; SE, skipped exon; MXE, mutually exclusion exons; A5SS, alternative 5’ splice site; A3SS, alternative 3’ splice site; RI, retained intron.

Functional enrichment analysis revealed that the genes in human dCD8 T cells showing upregulated AS events or downregulated AS events (when compared with autologous pCD8 T cells) were enriched in the GO terms involved in cellular metabolic process and organelle organization (Figure S10 in Supplementary Material). Furthermore, the genes containing upregulated or downregulated AS events uniquely (upregulated: 1,338 genes; downregulated: 1,538 genes), or those showing evidence of both upregulated and downregulated AS events simultaneously (1,178 genes), were remarkably enriched in the GO category related to cellular metabolic process (Figure 7). Additionally, when AS events were subgrouped into SE, MXE, A5SS, and A3SS, respectively, similar results were also observed (Figures S11–S18 in Supplementary Material). Collectively, these data showed that the human dCD8 T-cell genes undergoing upregulated or downregulated AS events are mainly related to cellular metabolic process.

### AS Is Not a Major Contributor to Gene Expression-Level Changes Between Human pCD8 and dCD8 T Cells

**Figure 8:**
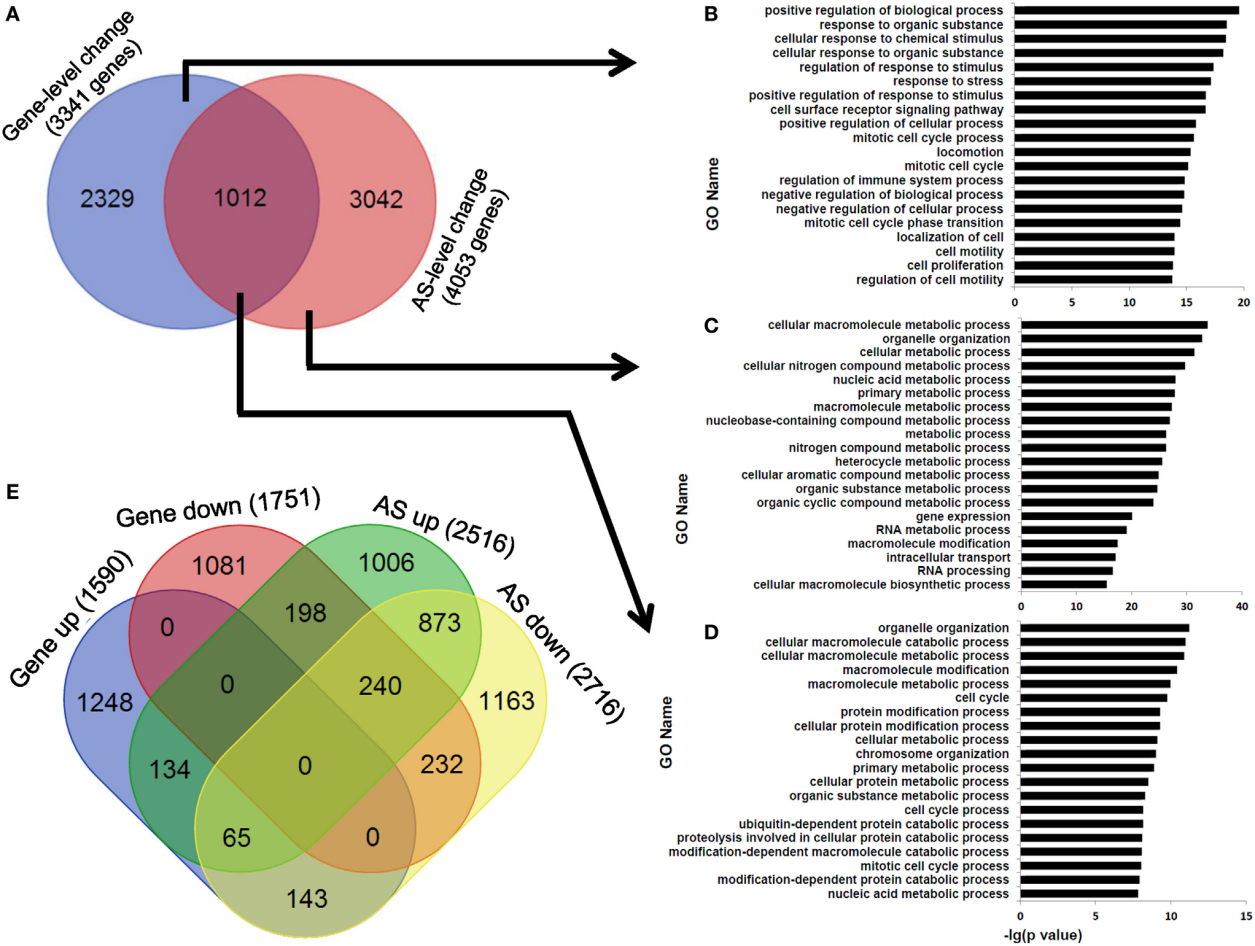
AS is not a major contributor to gene expression-level changes between paired pCD8 and dCD8 T cells. **(A–D)** Venn diagram **(A)** and functional enrichment analysis **(B–D)** for the genes showing significant AS-level and expression-level changes between in human dCD8 and pCD8 T cells. The top 20 GO terms (biological process) were indicated **(B–D)**. **(E)** Venn diagram of genes showing AS-level and/or expression-level upregulation and/or downregulation, in dCD8 T cells with respect to autologous pCD8 T cells. The five types of AS events including SE, MXE, A5SS, A3SS, and RI were combined together. Abbreviations: AS, alternative splicing; up, upregulated; down, downregulated; pCD8 T, peripheral blood CD8^+^ T; dCD8 T, decidual CD8^+^ T; GO, gene ontology; SE, skipped exon; MXE, mutually exclusion exons; A5SS, alternative 5’ splice site; A3SS, alternative 3’ splice site; RI, retained intron.

At last, we observed that the overlapping ratio between the sets of genes showing differential AS-level and expression-level changes was low: 1,012 genes in the overlap versus 3,341 genes showing expression-level difference and 4,053 genes showing AS-level difference between human dCD8 and pCD8 T cells (Figure 8A). Functional enrichment analysis revealed that the genes undergoing both expression- and AS-level changes were prominently enriched in the GO terms related to organelle organization and cellular metabolic process, which were different from those genes undergoing only expression-level change but similar to those showing only AS-level change (Figures 8A–D). Since the downregulated genes were also remarkably enriched in cellular metabolic process in human dCD8 T cells (Figure 2), we conjectured that AS might have contributed to the regulation of their expression. However, we observed that the overlapping ratio between sets of genes showing AS-level and expression-level downregulations was surprisingly low as well: 232 genes in the overlap versus 1,751 genes undergoing expression-level downregulation and 2,716 genes undergoing AS-level downregulation (Figure 8E); very low ratio was also found between sets of genes showing both AS- and expression-level upregulation (Figure 8E). Together, these results indicated that AS is not a major contributor to gene expression-level changes observed between human pCD8 and dCD8 T cells.

## Discussion

Since only half of the fetal genes are derived from the mother, the developing embryo and placenta are commonly regarded as a “semi-allogeneic graft” (34). The composition and role of the immune cells that populate the decidua are highly specialized, not merely to facilitate embryonic development and foster placental function, but also to minimize the possibility that the fetus is attacked as a foreign allograft and to provide protective immunity against the invading pathogens (2). Maternal CD8^+^ T cells are the principal candidates to recognize and respond to fetal HLA antigens in the decidua where dCD8 T cells are the most abundant lymphocytes during early human pregnancy (2, 4, 5). Although the activity of dCD8 T cells has been thought to be delicately tempered during healthy pregnancy, the phenotypic and functional characteristics of these cells are still largely unknown and remain controversial (4, 5, 7, 8, 10–12).

In this study, we have deep surveyed the genome-wide transcriptional difference between paired dCD8 and pCD8 T cells during human early pregnancy using high-throughput mRNA-Seq. Our data showed that human dCD8 T cells highly upregulated the genes related to immune effector process, M phase of mitotic cell cycle, antigen processing and presentation, T-cell activation, and cell proliferation/division (Figures 2 and 4; Figures S3 and S4 in Supplementary Material), indicating that these cells are endowed with elevated activation, high-proliferation potential, and enhanced immune response. Indeed, our flow cytometry data revealed that human dCD8 T cells dramatically upregulated the protein levels of activation antigens CD69, CD38, CD122, CD276, and ICOS (Figure 3). In addition, most of the MHC-II genes were expressed at a higher level in human dCD8 than pCD8 T cells (Figure S19 in Supplementary Material). Furthermore, more intracellular cytokines IFN-γ and IL-17A, and degranulation antigen CD107a, were produced in human dCD8 T cells upon stimulation with PMA and ionomycin *in vitro* (Figure 4), indicating that these cells have an enhanced functionality on a per-cell basis. Therefore, our findings suggested that human dCD8 T cells during healthy early pregnancy are fully functional with increased activation and proliferation rather than being suppressed, which are in disagreement with the hypothesis that dCD8 T cells lack complete activation and fail to acquire robust effector functions (10, 13, 35, 36), but are consistent with the results showing that dCD8 T cells are regionally activated and functionally capable of rapid proliferation, cytolysis, and cytokine production (7, 12, 20, 37, 38).

CD4^+^ Treg cells, which are concentrated at the maternal–fetal interface and expanded in the peripheral blood and decidua of pregnant mammals, play critical roles in the creation of a privileged tolerant microenvironment, and immunological acceptance of the semi-allogeneic fetus (1, 39). Recently, growing evidences suggest that CD8^+^CD122^+^PD-1^+^ T cells are also a subset of Treg cells, which have more ability to suppress the allograft rejection and undergo faster homeostatic proliferation than conventional CD4^+^CD25^+^Foxp3^+^ Treg cells (31, 32, 40). Our data showed that human dCD8 T cells exhibit a high-proliferative capacity and CD8^+^CD122^+^PD-1^+^ Treg cells are largely concentrated in early pregnancy decidua (Figure 4). However, unlike our previous finding in dCD4 T cells (18), Foxp3^+^ cells were extremely few in both human pCD8 and dCD8 T cells (Figure S20 in Supplementary Material), indicating that decidual CD8^+^ and CD4^+^ T cells display a different Treg-cell phenotype. Shao et al. revealed that human placental trophoblasts can activate a subset of CD8^+^ Treg cells expressing the mucosal markers CD101 and CD103 (6), and Elena Uss et al. reported that CD103 is also a marker for alloantigen-induced CD8^+^ Treg cells producing a considerable amount of the immunosuppressive cytokine IL-10 (33). Here, the genes encoding CD101, CD103, and IL-10 were observed to be prominently upregulated in human dCD8 T cells (Figures S7 and S19 in Supplementary Material). In addition, a significantly higher percentage of CD8^+^CD28^−^ (suppressor) T cells that express CD103 but not perforin, was identified in human decidua than peripheral blood during early pregnancy (4, 41). Therefore, our data together with previous studies (6, 33, 41, 42) suggested that human dCD8 T cells have a CD8-Treg phenotype and play an important role in local immune tolerance toward the fetus. However, it is still unclear whether the increased CD8^+^ Treg cells in human early decidua are the outcome of a local expansion and differentiation or whether they migrate from the periphery.

Memory is a distinguishing feature of adaptive immune response, and memory CD8^+^ T cells are commonly divided into two distinct subsets: central memory T (T_CM_) cells and T_EM_ cells (28, 43). Similar to other mucosal tissues, the CD8^+^ T cells residing at human endometrium and decidua have been demonstrated to display a CD8-T_EM_ phenotype (4, 44). In addition, previous studies have shown that T_CM_ cells are a long-lived and stem cell-like memory subset, while T_E_ and T_EM_ cells are the first responders capable of possessing immediate effector functions and are undergoing extensive proliferation (34, 45). Our present data (Figures 4E–G) and previous study (18) revealed that human dCD8 T cells mainly consist of T_EM_ and effector T (T_E_) cells whereas dCD4 T counterparts mainly contain T_EM_ and T_CM_ cells; however, pCD8 T cells significantly increase the proportion of T_N_ cells but decrease T_EM_ cells (Figures 4E–G). Therefore, our data indicated that human dCD8 T cells have a higher rate of proliferation than dCD4 and pCD8 T counterparts. Indeed, both GO annotation and GSEA analysis revealed that the upregulated genes in human dCD8 T cells were most significantly enriched in the terms related to M phase of mitotic cell cycle (Figure 2), and flow cytometry data also showed that the proportion of dCD8 T cells is higher than pCD8 and dCD4 T cells (Figure 1). Increased human dCD8 T cells might contribute to immune protection against the invading pathogens and to tolerance toward the growing semi-allogeneic fetus.

Moreover, both CD69 and CD103, that can prevent cell egress and are expressed at a higher level by mucosal memory T cells than circulating memory T cells (44, 46, 47), were observed to be upregulated in human dCD8 cells (Figure 3; Figures S6 and S7 in Supplementary Material), indicating that these cells have a “tissue-resident effector-memory T cell” (T_REM_) phenotype during human early pregnancy. Although memory dCD8 T cells were hypothesized to be virus-specific T cells to protect the fetus against invading pathogens in uncomplicated pregnancy (48), the definite role and specificity of these dCD8 T_REM_ cells remain largely unknown and need to be determined by more experiments.

High-throughput mRNA-Seq allows not only to quantify and differential analyze the gene expression, but also to identify the cell type-specific novel/new splice junctions and AS events, at a genome-wide level with a high reliability and sensitivity *via* millions of short cDNA fragments (14, 15, 24). Blencowe and colleagues performed the first analysis of AS complexity in human six tissues using high-throughput mRNA-Seq datasets, and reported that the new splice junctions were identified in 18.8–24.1% of the multiexon genes, many of which are tissue specific (15). Besides, Rendon and colleagues detected 7,881 novel splice junctions, which are enriched in the genes related to critical cellular processes and use a high ratio of the non-canonical splicing motif GC-AG, by deep sequencing the mRNA from eight primary human hematopoietic cells (24). Similarly, 6,322 novel splice junctions (belonging to 2,137 genes) were identified in human dCD8 and pCD8 T cells, analysis of which highlighted the genes involved in immune system, and a significantly high proportion of the splice motif GC-AG (Figure 5; Figure S8 in Supplementary Material). Though both the splice sites GT-AG and GC-AG are recognized and processed by the classic U2-type splicesome, the splice motif GC-AG tends to be alternatively spliced (24).

Previous studies revealed that there are ~100,000 AS events in major human tissues with middle- to high abundance (15), and more than half of human genes are subject to be alternative spliced (49). Likewise, we identified a total of 142,132 AS events, belonging to 10,556 genes, in human decidual and peripheral blood CD8^+^ T cells (Figure 6). In addition, our data showed that the AS events are ranked from the most abundant to the less in the order: SE (72,538 events), A3SS (26,953 events), MXE (24,102 events), A5SS (16,413 events), and RI (2,126 events) (Figure 6), agreeing with the previous study showing that there is a higher fraction of RI events in invertebrates whereas SE events are more prevalent in vertebrates (8). These results indicated that the high rates of exon shuffling and protein-domain rearranging in vertebrate cells might correlate with the high level of SE regulation (8, 50).

The transitions between activation and quiescence in T cells require apportioning adequate nutrients into different pathways, which are regulated by metabolic processes (45). Herein, we found that there is a comparable number of upregulated and downregulated AS events in human dCD8 T cells (versus paired pCD8 T cells), both of which are enriched in the genes related to cellular metabolic process (Figures 6 and 7). These data indicated that these differentially expressed AS events are indeed functional but not used in a tissue-specific manner. Moreover, in agreement with previous studies (18, 51, 52), our data revealed that AS event is not a major contributor to the gene expression-level changes observed between paired pCD8 and dCD8 T cells (Figure 8). Currently, the PacBio RS sequencing platform, which uses the third-generation long-read technology, can capture the full-length transcript isoforms and alleviate the deficit of transcriptome assembly on the basis of high-throughput short reads obtained from Illumina platform (24, 50, 53). Therefore, it is very interesting to investigate the connections of the differentially expressed AS events with the expression of different transcript isoforms and with the physiological role of early dCD8 T cells in healthy pregnancy. These connections are also great important for grasping human dCD8 T-cell biology.

Surgical abortion by vacuum aspiration has become a safe, effective, and widely used method to terminate the first-trimester pregnancy (54). Harsem et al. found that vacuum aspiration is a new way to obtain sufficient decidual tissues to study the function and morphology of extravillous trophoblast cells (55), and a growing number of researchers have applied this method to collect decidual samples to investigate the composition, phenotype or function of immune cells in human pregnancy decidua (18, 22, 56–59). Although we also obtained the decidual tissues by vacuum aspiration and then placed them in sterile ice-cold PBS immediately, further work is needed to resolve this issue: whether surgical abortion by vacuum aspiration could distort dCD8 T-cell phenotype and function away from the natural *in vivo* status. Moreover, to maintain the viability of dCD8 T cells as much as possible, separation of DMCs from human decidua was performed using the procedure of non-enzymatic leukocytes treatment, as many previous studies described (12, 18–22); and purification of these cells was achieved by FACS sorting, immediately. All these experiments were performed continuously and finished within 1 day. However, there was still a limitation that we did not use a LIVE/DEAD dump in these flow experiments to exclude the dead cells completely.

As mentioned above, our data showed that human pCD8 T cells consist mainly of T_N_ and T_E_ cells, and dCD8 T cells mainly contain T_E_ and T_EM_ cells whereas T_N_ cells are almost absent (Figures 4E–G). In addition, both the local and systemic maternal immune systems have been proved to be modulated during human early pregnancy to maintain immune tolerance (38). Therefore, a better study is to isolate the CD8^+^ T_E_ cells in human pregnancy decidua and peripheral blood as well as in non-pregnant endometrium and periphery, and to compare the transcriptional and AS landscapes among these cells.

Taken together, our data revealed that although the numbers of DEGs are comparable in human dCD8 versus pCD8 T cells, dCD8 T cells highly upregulate the genes involved in M phase of mitotic cell cycle and immune response but downregulate those related to metabolic process. Moreover, dCD8 T cells show increased activation and proliferation, display CD8-Treg and effector-memory phenotypes as well as are endowed with an enhanced functionality on a per-cell basis. Additionally, the novel splice junctions use a high ratio of the non-canonical splicing motif GC-AG and are enriched in the genes involved in immune response; and there is a comparable number of upregulated and downregulated AS events, both of which are enriched in the genes related to cellular metabolic process, in human dCD8 T versus pCD8 T cells. Finally, AS is not a major contributor to the gene expression-level changes observed between paired pCD8 and dCD8 T cells. Our study thus provides a comprehensive framework of the transcriptional and AS landscapes as well as reveals the functional feature of human early dCD8 T cells, which deepens our understanding of the biology of these cells and the physiology of healthy early pregnancy. Since growing evidences have shown that the alteration in human dCD8 T-cell number, activation or function, is associated with the pathophysiology underlying pregnancy-related complications such as spontaneous abortion (32, 60), preeclampsia (39, 43, 61), hydatidiform mole (27, 30), and anembryonic pregnancy (19, 20), further studies are needed to investigate the roles of dCD8 T cells in the pathogenesis of these human complications as well as poor postnatal health.

## Ethics Statement

The study was approved by the Medical Ethics Committee of the International Peace Maternity and Child Health Hospital of China Welfare Institute and all experiments were performed according to the principles of the Declaration of Helsinki. Informed consent was assigned individually from all participants before enrollment.

## Author Contributions

WZ designed and performed most of the experiments, and drafted the manuscript. XL, ZL, YZhe, TY, XL, JZ, SZ, XM, X-RL, and XQ performed a part of the experiments. SF analyzed a majority of the mRNA-Seq data. WZ, XL, ZL, and AK edited the manuscript. WZ, YZha, FT, and YL supervised the study and proofread the manuscript.

## Conflict of Interest Statement

The authors declare that the research was conducted in the absence of any commercial or financial relationships that could be construed as a potential conflict of interest.

## Abbreviations

AS, alternative splicing; mRNA-Seq, mRNA sequencing; dCD8 T, decidual CD8^+^ T; pCD8 T, peripheral blood CD8^+^ T; Treg, T regulatory; DEGs, differentially expressed genes; BP, biological process; PBMC, peripheral blood mononuclear cells; GO, gene ontology; KEGG, kyoto encyclopedia of genes and genomes; GSEA, gene set enrichment analysis; FDR, false discovery rate; NES, normalized enrichment score; SE, skipped exon; MXE, mutually exclusion exons; A5SS, alternative 5’ splice site; A3SS, alternative 3’ splice site; RI, retained intron.
