# Supplementary material for: Deep Surveying of the Transcriptional and Alternative Splicing Signatures for Decidual CD8+ T Cells at the First Trimester of Human Healthy Pregnancy
Source: Front Immunol. 2018 May 4;9:937. doi: 10.3389/fimmu.2018.00937 (PMC5946033; doi:10.3389/fimmu.2018.00937)
Supplement: Supplementary file 1 [file Image_1.PDF]

Supplementary Materials

Title: Deep Surveying of the Transcriptional and Alternative Splicing Signatures for Decidual CD8$^+$ T Cells at the First Trimester of Human Healthy Pregnancy

Authors:

Weihong Zeng[1,#], Xinmei Liu[1,#], Zhicui Liu[2,#], Ying Zheng[3], Tiantian Yu[1], Shaliu Fu[4], Xiao Li[1], Jing Zhang[1], Siming Zhang[1], Xiaoling Ma[1], Xiao-Rui Liu[1], Xiaoli Qin[1], Asma Khanniche[5], Yan Zhang[6,*], Fuju Tian[1,*] and Yi Lin[1,*]

Institution:

[1] Institute of Embryo-Fetal Original Adult Disease Affiliated to Shanghai Jiao Tong University School of Medicine, the International Peace Maternity & Child Health Hospital, Shanghai Jiao Tong University School of Medicine, Shanghai 200030, P. R. China.

[2] Department of Dermatology, Shanghai Tenth People's Hospital, Tongji University School of Medicine, Shanghai 200072, P. R. China..

[3] Out-patient Operatingroom, the International Peace Maternity & Child Health Hospital, Shanghai Jiao Tong University School of Medicine, Shanghai 200030, P. R. China.

[4] School of Life Science, Tongji University, Shanghai 200092, P. R. China.

[5] Shanghai Institute of Immunology, Shanghai Jiao Tong University School of Medicine, Shanghai 200025, P. R. China.

[6] Department of Obstetrics and Gynecology, Renmin Hospital of Wuhan University, Wuhan 430060, P. R. China.

[#] These authors contributed equally to the study.

[*] **Corresponding author:**

Yan Zhang, Department of Obstetrics and Gynecology, Renmin Hospital of Wuhan University, Wuhan 430060, P. R. China. Telephone: +86-27-88041911. Fax:

+86-27-88041911. E-mail: zyan2200@gmail.com.

Fuju Tian, Institute of Embryo-Fetal Original Adult Disease Affiliated to Shanghai Jiao Tong University School of Medicine, the International Peace Maternity & Child Health Hospital, Shanghai Jiao Tong University School of Medicine, No. 910, Hengshan Road, Shanghai 200030, P. R. China. E-mail: tianfuju2012@126.com.

Yi Lin, Institute of Embryo-Fetal Original Adult Disease Affiliated to Shanghai Jiao Tong University School of Medicine, the International Peace Maternity & Child Health Hospital, Shanghai Jiao Tong University School of Medicine, No. 910, Hengshan Road, Shanghai 200030, P. R. China. Telephone: +86-21-64070434. Fax: +86-21-64073421. E-mail: yilinonline@126.com.

Running title: Transcriptome of human decidual $CD8^+$ T cells

Supplementary Figures

A

Group 1: Samples for mRNA-Seq

Sample	Age (years)	Gestational time (Days)
1	28	47
2	22	44
3	27	58

B

Group 2: Samples for validation of mRNA-Seq data and evaluation of CD8-Treg frequency

Sample	Age (years)	Gestational time (Days)
1	39	42
2	28	50
3	28	50
4	33	38
5	22	42

C

Group 3: Samples for detection of IFN-γ and IL-17A secretion and memory phenotype in CD8⁺ T cells

Sample	Age (years)	Gestational time (Days)
1	35	43
2	34	50
3	39	43
4	30	43

D

Group 4: Samples for detection of CD107a expression in CD8⁺ T cells

Sample	Age (years)	Gestational time (Days)
1	25	59
2	26	46
3	33	44
4	19	75
5	22	67

Figure S1. Summary of the information regarding the twenty-seven healthy women at the first trimester of pregnancy recruited for this study. (A-B) Samples from three women (mean age 26 years, range 22–28 years; mean Gestational Day 50, range 44–58 days) were used for high-throughput mRNA sequencing (mRNA-Seq; A), and five others (mean age 30 years, range 22–39; mean gestational day 45, range 38–50) were enrolled to validate the mRNA-Seq data and evaluate CD8-Treg frequency (B). **(C-D)** Samples from four women (mean age 34 years, range 30–39; mean gestational day 45, range 43–50 days) were used to determine the IFN-γ and IL-17A secretion and memory phenotype (C), and five others (mean age 25 years, range 19–33; mean gestational day 58, range 44–75) were applied to evaluate CD107a expression in $CD8^+$ T cells (D).

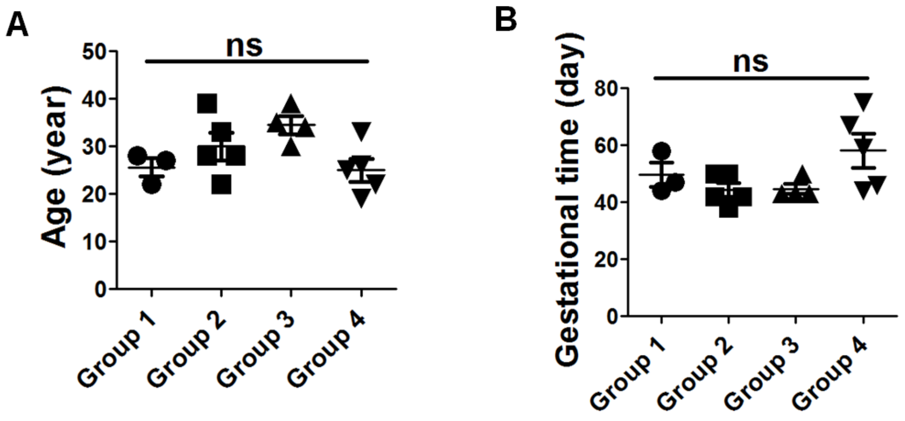

Figure S2. The differences in both age and gestational day are not statistically significant across the four cohorts recruited in this study. The normality of age and gestational day in Figure S1 was evaluated by Kolmogorov-Smirnov test, which indicated that the data were not normally distributed. Therefore, statistical analysis was performed using Kruskal-Wallis test. Analysis revealed that the difference in both age and gestational day were not statistically significant across these four cohorts (P-value = 0.0539 in age, and 0.1177 in gestational day).

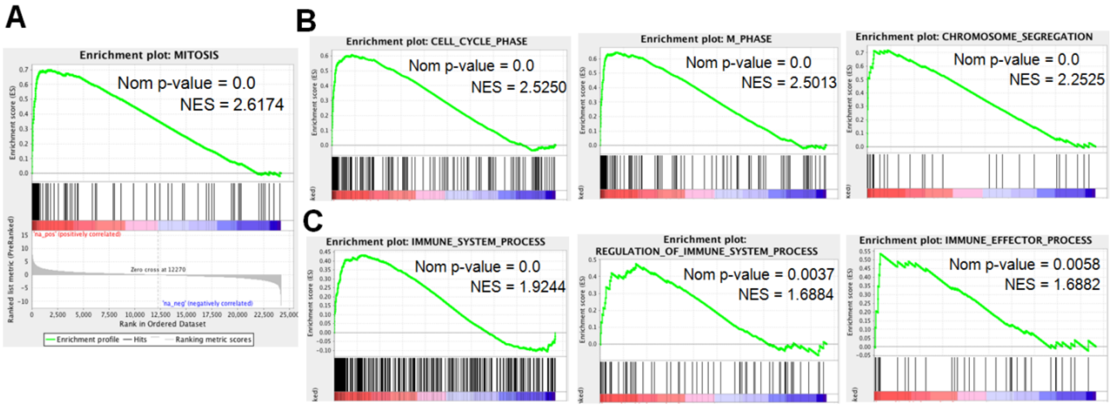

Figure S3. GSEA plots of gene sets in GO categories related to M phase of mitotic cell cycle and immune response in human dCD8 *versus* pCD8 T cells. The NES reflects the degree to which a gene set is upregulated (positive NES) in dCD8 T cells and the corresponding nom p-value is indicated. GSEA, Gene Set Enrichment Analysis; GO, Gene Ontology; dCD8 T, decidual CD8+ T; pCD8 T, peripheral blood CD8+ T; Nom, Nominal; NES, Normalized Enrichment Score.

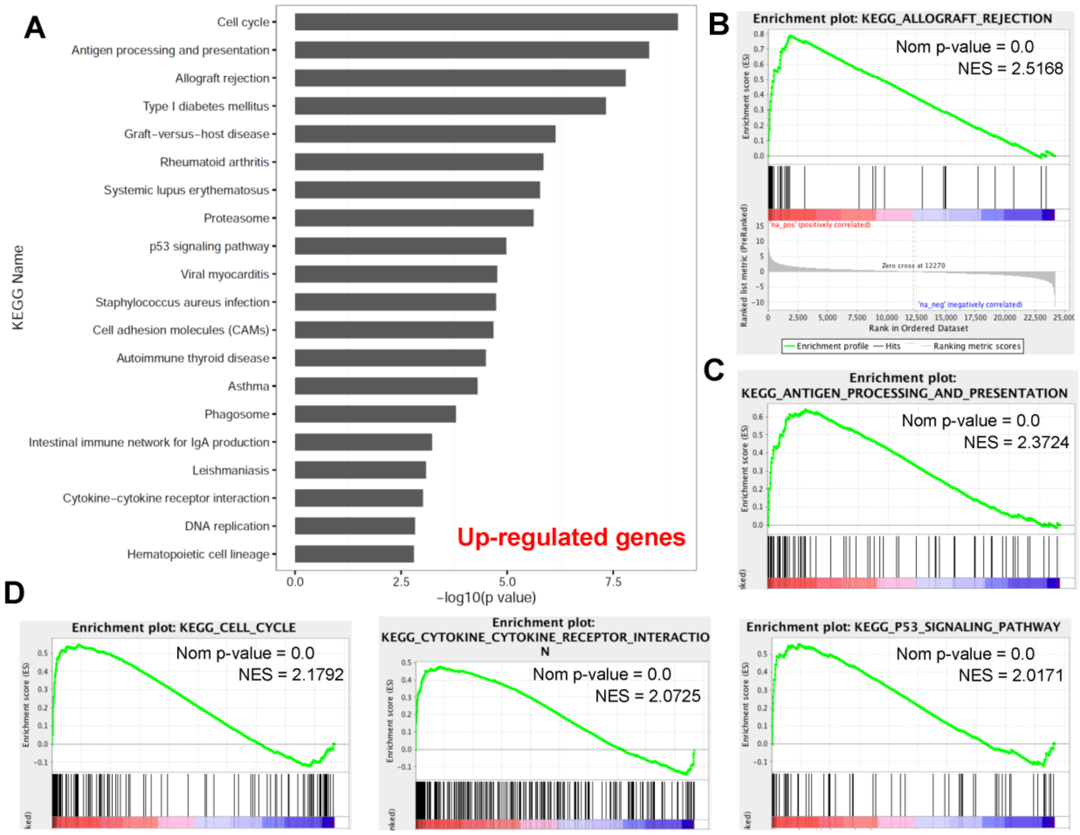

Figure S4. Human dCD8 T cells upregulate the genes involved in cell cycle process and immune system process. (A) The top 20 KEGG terms enriched for the upregulated genes in human dCD8 *versus* pCD8 T cells. **(B-D)** GSEA plots of gene sets in KEGG categories including allograft rejection (B), antigen processing and presentation (C), cell cycle, cytokine/cytokine receptor interaction and p53 signaling pathway (D) in human dCD8 *versus* pCD8 T cells. The NES reflects the degree to which a gene set is upregulated (positive NES) in dCD8 T cells and the corresponding nom p-value is indicated. KEGG, Kyoto Encyclopedia of Genes and Genomes; GSEA, Gene Set Enrichment Analysis; Nom, Nominal; NES, Normalized Enrichment Score; dCD8 T, decidual CD8$^+$ T; pCD8 T, peripheral blood CD8$^+$ T.

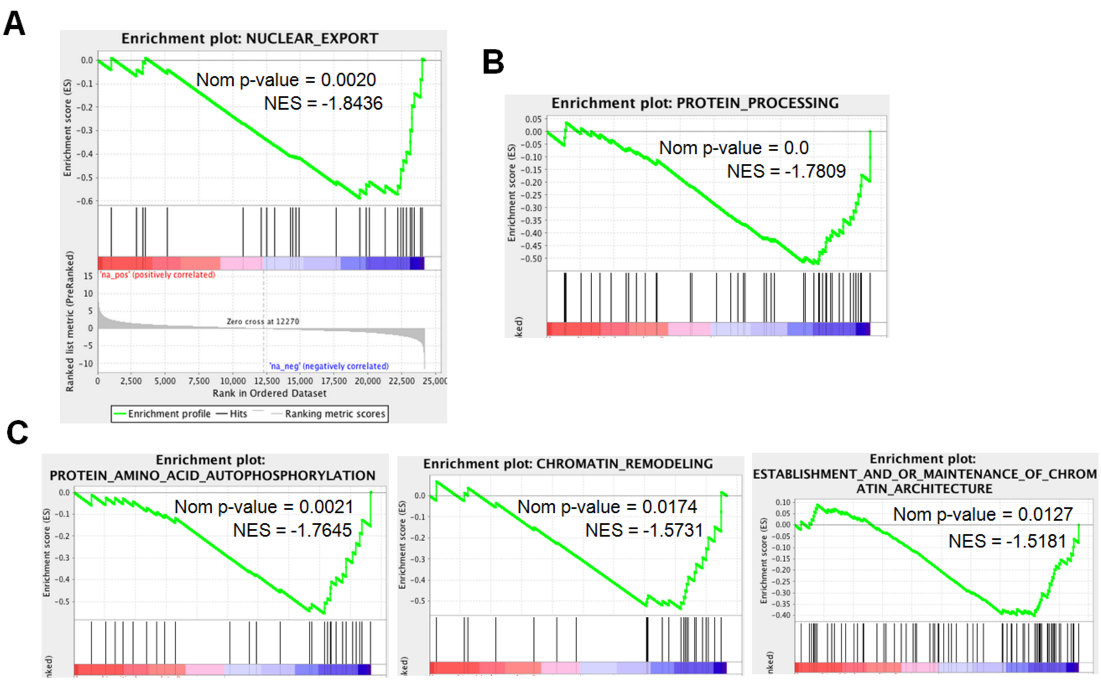

Figure S5. GSEA plots of gene sets in GO categories related to metabolic process in human dCD8 *versus* pCD8 T cells. The NES reflects the degree to which a gene set is downregulated (negative NES) in dCD8 T cells and the corresponding nom p-value is indicated. GSEA, Gene Set Enrichment Analysis; GO, Gene Ontology; dCD8 T, decidual CD8$^+$ T; pCD8 T, peripheral blood CD8$^+$ T; Nom, Nominal; NES, Normalized Enrichment Score.

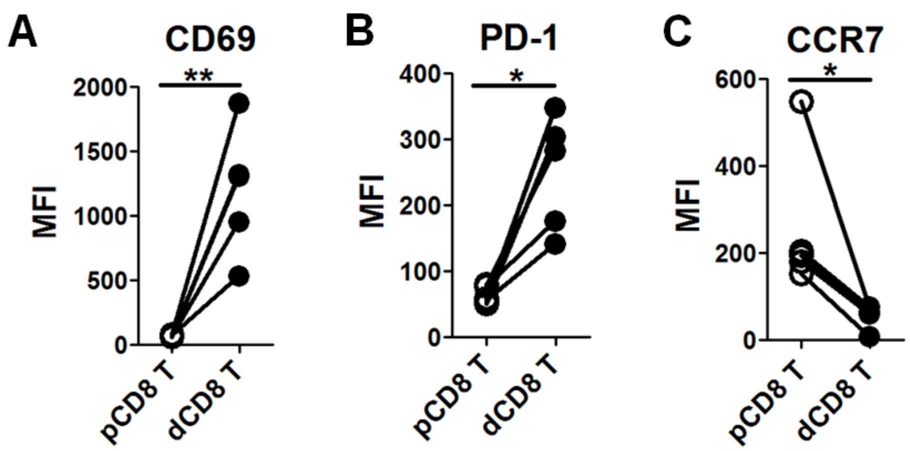

Figure S6. Human dCD8 T cells highly upregulate expression of CD69 and PD-1, but downregulate CCR7. Cumulative data illustrating the comparison of molecular expression, measured as the geometric MFI, of indicated proteins between paired pCD8 and dCD8 T cells (n = 5 per group). pCD8 T, peripheral blood $CD8^+$ T; dCD8 T, decidual $CD8^+$ T; MFI, mean fluorescent intensity.

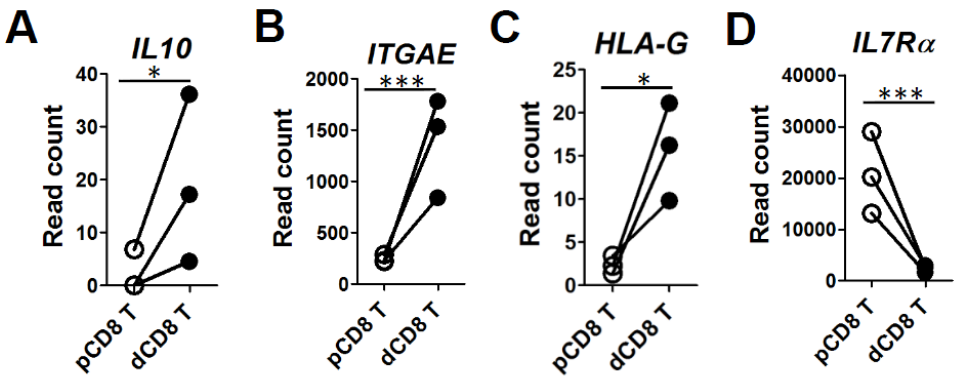

Figure S7. Comparison of the gene expression (measured as the read count) of *IL10*, *ITGAE*, *HLA-G* and *IL7Rα* between paired pCD8 and dCD8 T cells. Each symbol reflects a sample and each line reflects the samples from the same person (n = 3 per group). Differential expression analysis was performed using DESeq2 algorithm (paired test). dCD8 T, decidual CD8$^+$ T; pCD8 T, peripheral blood CD8$^+$ T.

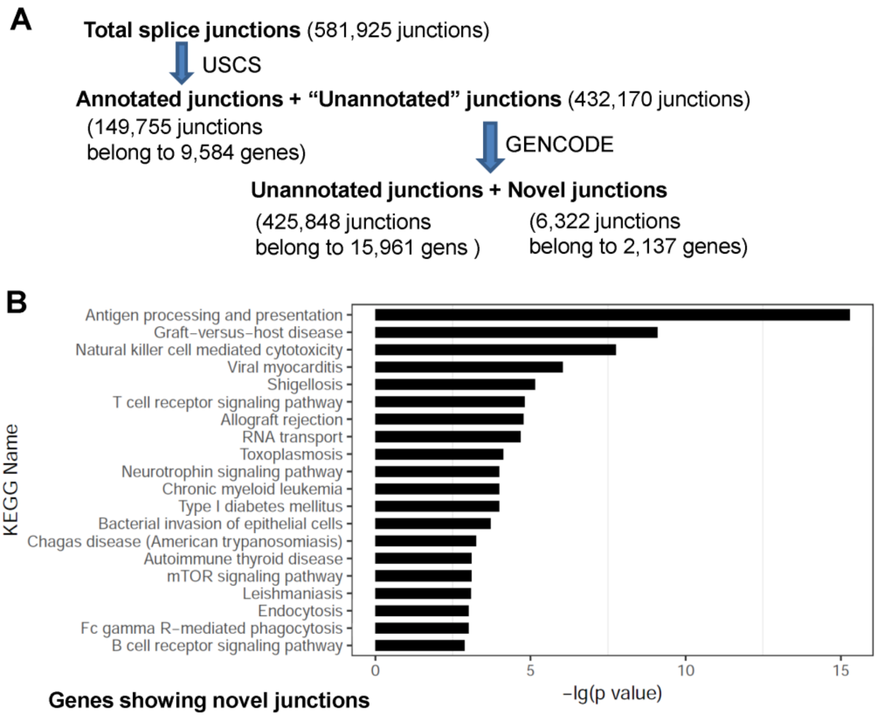

Figure S8. Novel splice junctions are enriched in the genes related to immune response. (A) Schematic diagram illustrating the strategy used to identify the novel splice junctions in human pCD8 and dCD8 T cells. Sequenced reads were aligned to the human reference genome (hg19 version) using the STAR software package and splice junctions were identified. A splice junction was defined as "unannotated" if not present in the University of California Santa Cruz (UCSC) expressed sequence tag (EST)/mRNA data set. These "unannotated" splice junctions were further compared to the GENCODE data set to identify the novel splice junctions. **(B)** The top 20 KEGG terms enriched for the genes showing novel splice junctions in human pCD8 and dCD8 T cells.

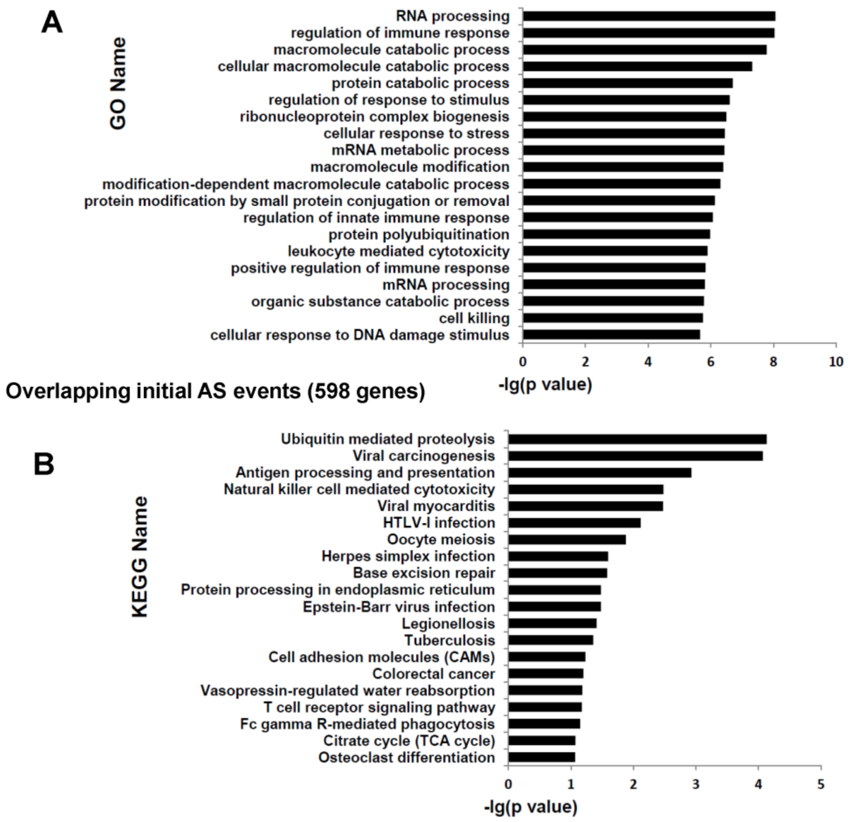

Figure S9. Functional enrichment analysis of the genes undergoing all five AS modes in human pCD8 and dCD8 T cells. (A-B) The top 20 GO (A) and KEGG (B) terms enriched for the genes undergoing all five AS modes (598 genes). AS, alternative splicing; GO, Gene Ontology; KEGG, Kyoto Encyclopedia of Genes and Genomes; pCD8 T, peripheral blood CD8^{+} T; dCD8 T, decidual CD8^{+} T.

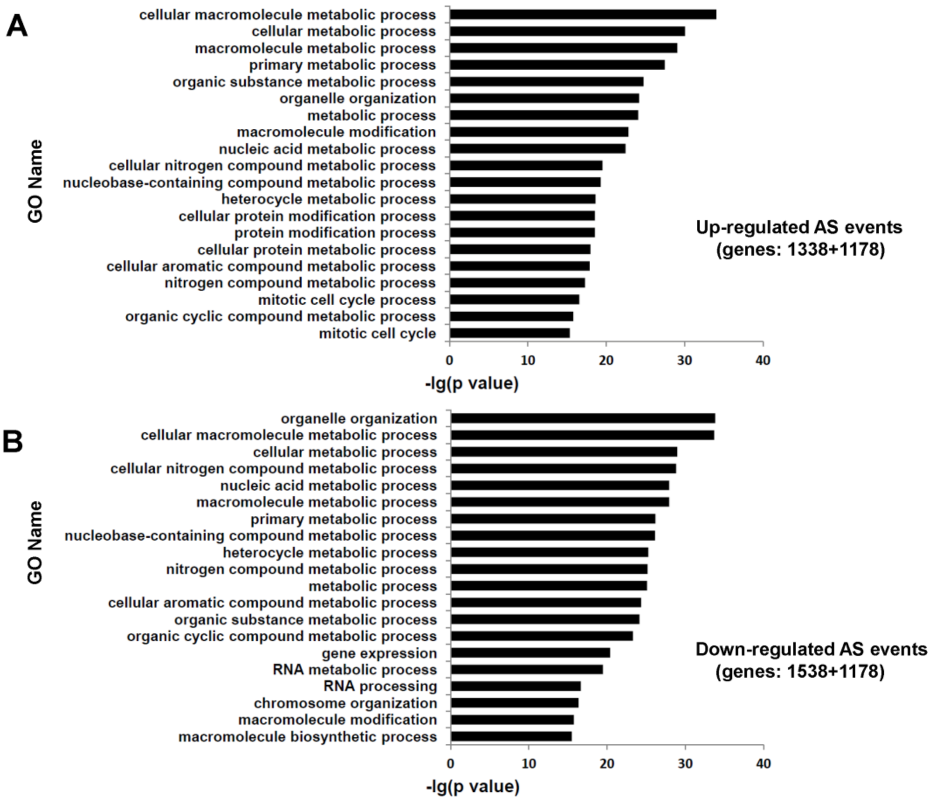

Figure S10. GO annotation for the genes undergoing upregulated or downregulated AS events in human dCD8 T cells. (A-B) The top 20 GO terms enriched for the genes undergoing upregulated (FDR < 0.05 with $\Delta\Psi > 0.05$ between samples; A) and downregulated (FDR < 0.05 with $\Delta\Psi < -0.05$ between samples; B) AS events (SE, MXE, A5SS, A3SS and RI are combined together) in human dCD8 T cells with respect to pCD8 T cells. GO, Gene Ontology; AS, alternative splicing; FDR, false discovery rate; SE, skipped exon; MXE, mutually exclusion exons; A5SS, alternative 5' splice site; A3SS, alternative 3' splice site; RI, retained intron.

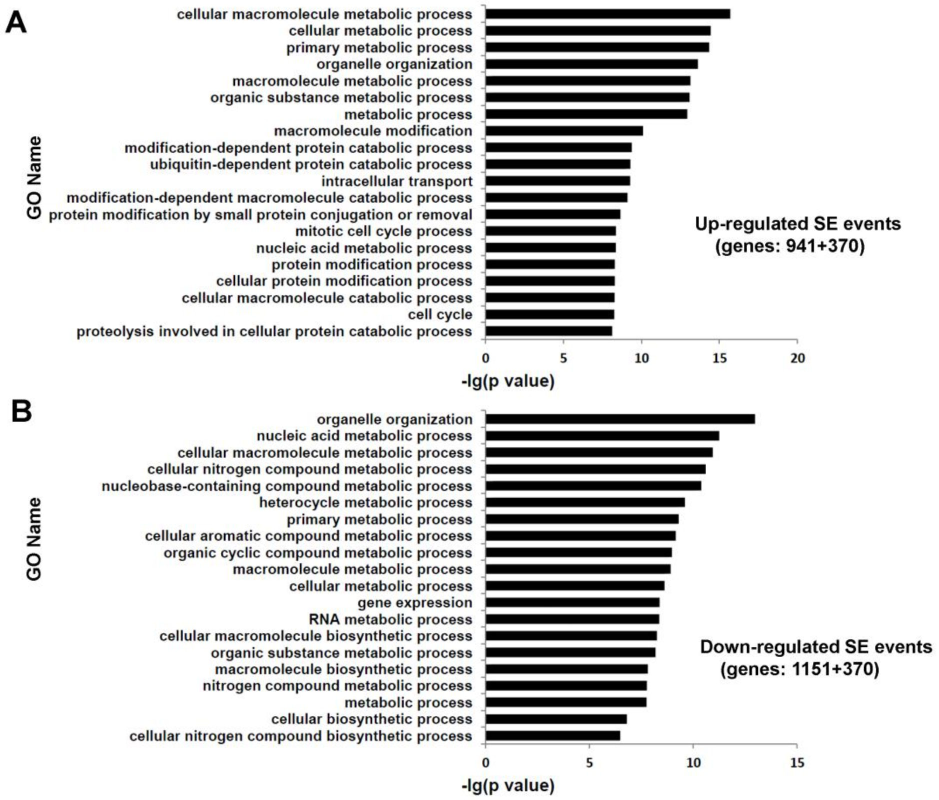

Figure S11. GO annotation for the genes undergoing upregulated or downregulated SE events in human dCD8 T cells. (A-B) The top 20 GO terms enriched for the genes undergoing upregulated (FDR < 0.05 with ΔΨ > 0.05 between samples; A) and downregulated (FDR < 0.05 with ΔΨ < -0.05 between samples; B) SE events in human dCD8 T cells with respect to pCD8 T cells. GO, Gene Ontology; SE, skipped exon; FDR, false discovery rate.

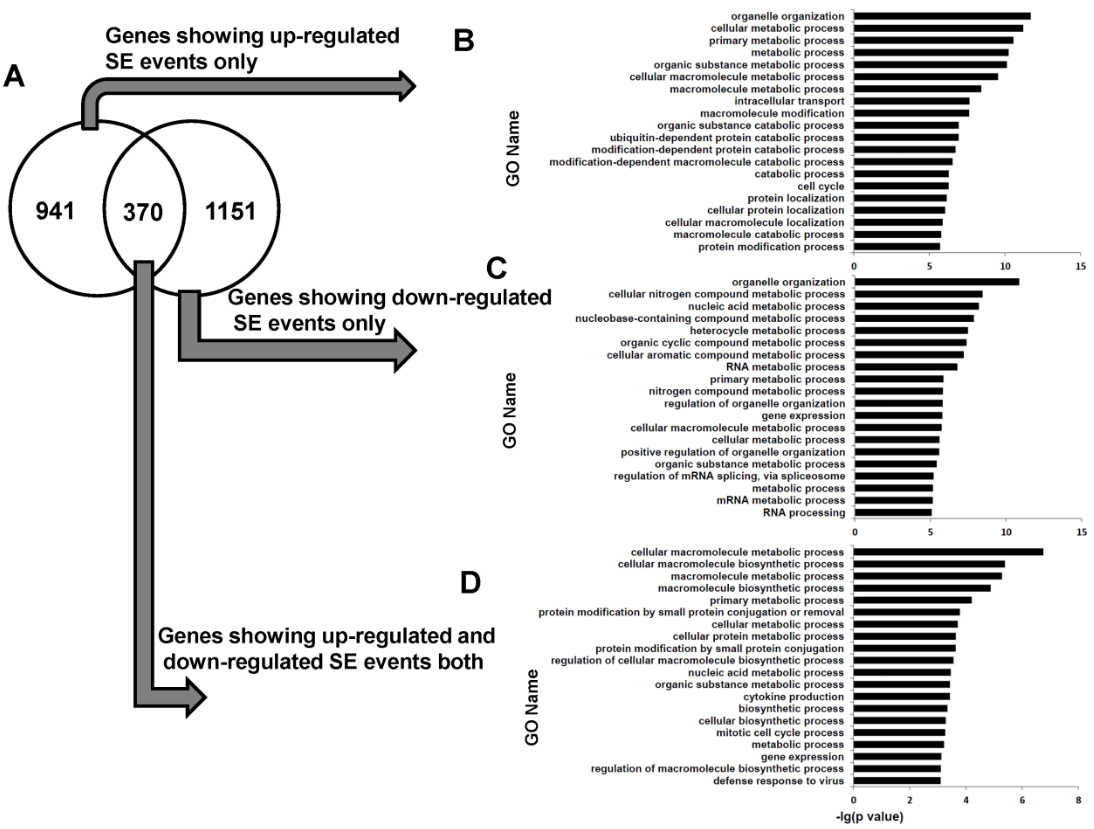

Figure S12. Venn diagram and functional enrichment analysis for the genes showing upregulated and downregulated SE events in human dCD8 T cells. (A-D) Venn diagram (A) and GO annotation (B-D) for the genes showing upregulated (FDR < 0.05 with ΔΨ > 0.05) and downregulated (FDR < 0.05 with ΔΨ < -0.05) SE events in human dCD8 T cells with respect to autologous pCD8 T cells. The top 20 GO terms (biological process) were indicated (b-d). GO, Gene Ontology; FDR, false discovery rate; SE, skipped exon.

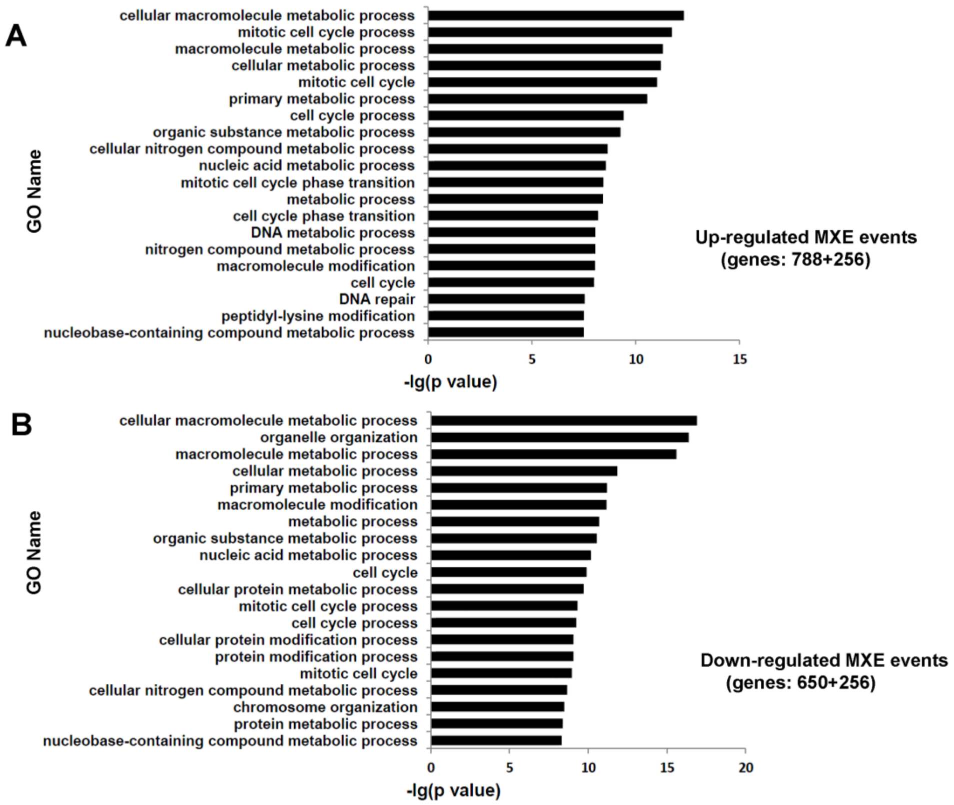

Figure S13. GO annotation for the genes undergoing upregulated or downregulated MXE events in human dCD8 T cells. (A-B) The top 20 GO terms enriched for the genes undergoing upregulated (FDR < 0.05 with $\Delta\Psi > 0.05$ between samples; A) and downregulated (FDR < 0.05 with $\Delta\Psi < -0.05$ between samples; B) MXE events in human dCD8 T cells with respect to pCD8 T cells. GO, Gene Ontology; MXE, mutually exclusion exons; FDR, false discovery rate.

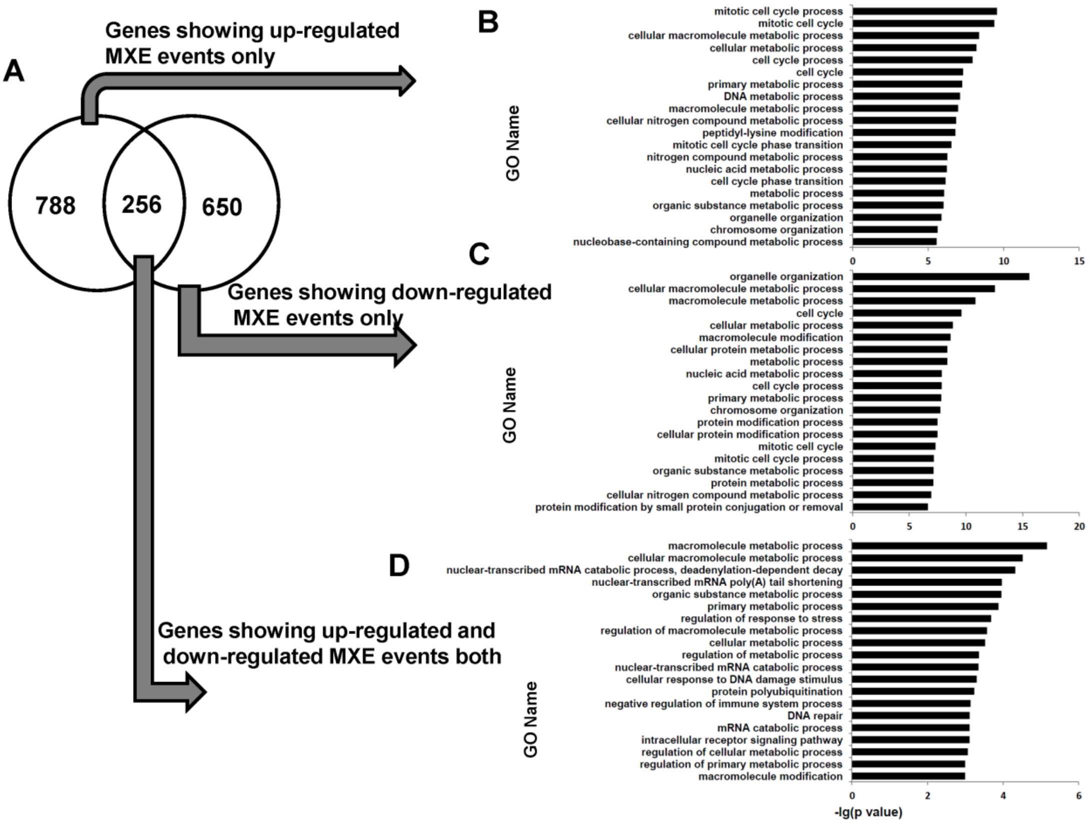

Figure S14. Venn diagram and functional enrichment analysis for the genes showing upregulated and downregulated MXE events in human dCD8 T cells. (A-D) Venn diagram (A) and GO annotation (B-D) for the genes showing upregulated (FDR < 0.05 with $\Delta\Psi > 0.05$) and downregulated (FDR < 0.05 with $\Delta\Psi < -0.05$) MXE events in human dCD8 T cells with respect to autologous pCD8 T cells. GO, Gene Ontology; MXE, mutually exclusion exons; FDR, false discovery rate.

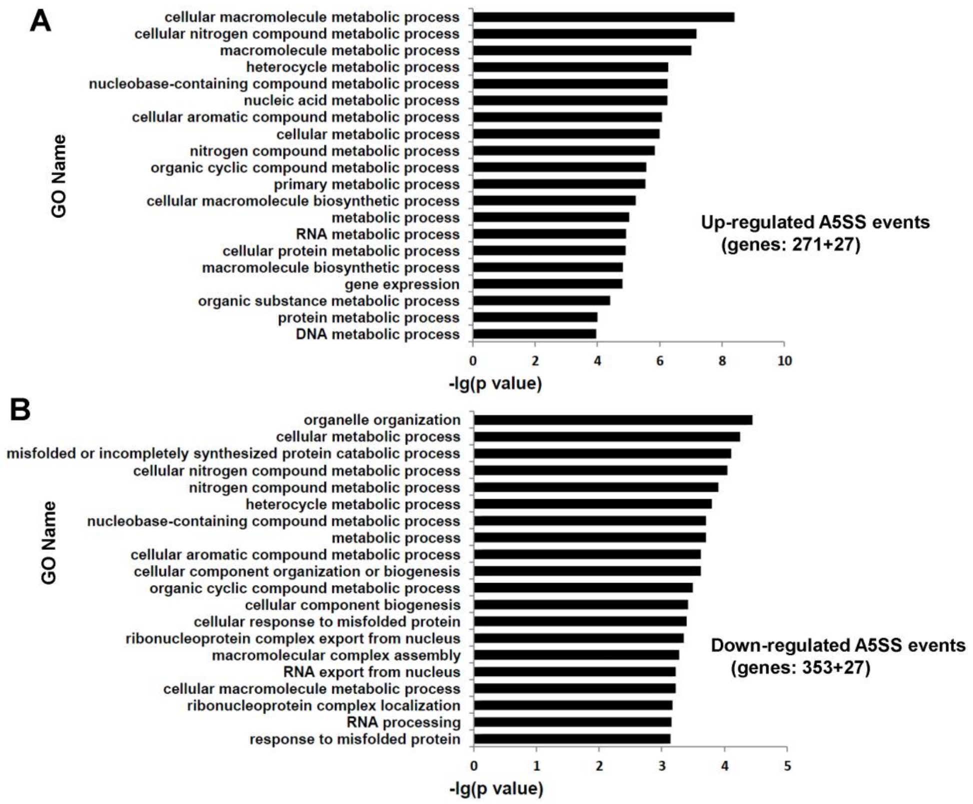

Figure S15. GO annotation for the genes undergoing upregulated or downregulated A5SS events in human dCD8 T cells. (A-B) The top 20 GO terms enriched for the genes undergoing upregulated (FDR < 0.05 with $\Delta\Psi > 0.05$ between samples; A) and downregulated (FDR < 0.05 with $\Delta\Psi < -0.05$ between samples; B) A5SS events in human dCD8 T cells with respect to pCD8 T cells. GO, Gene Ontology; A5SS, alternative 5' splice site; FDR, false discovery rate.

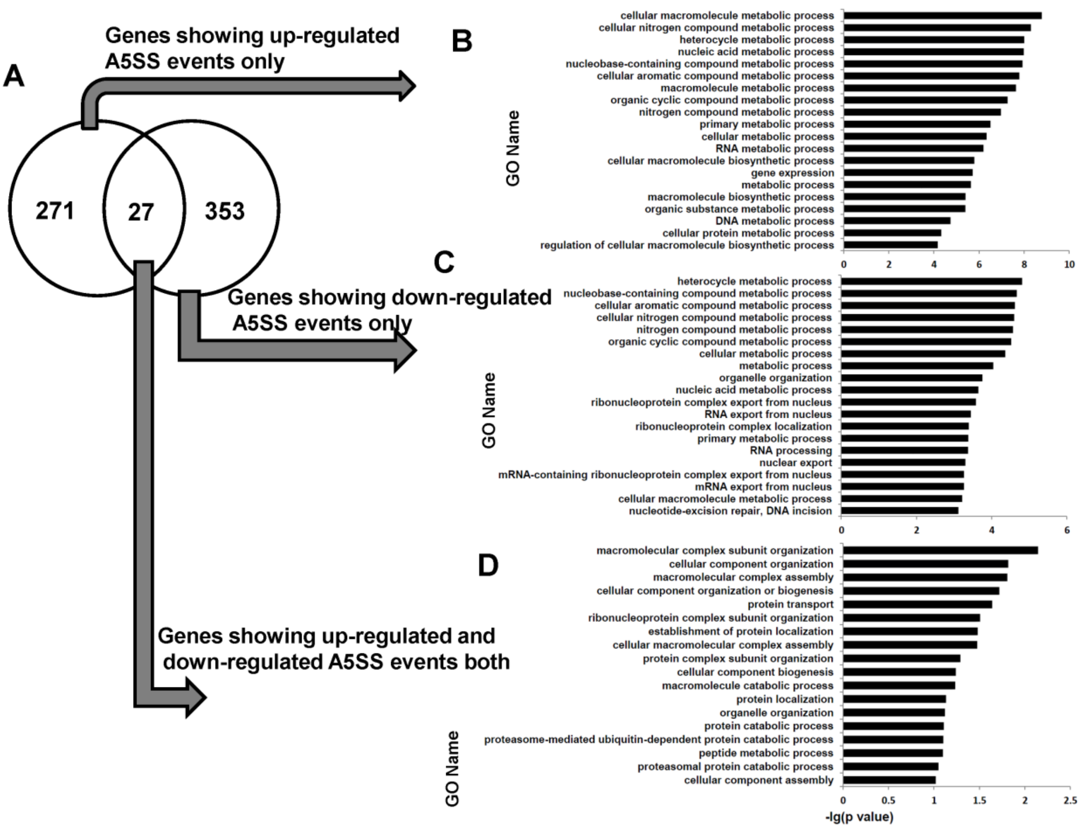

Figure S16. Venn diagram and functional enrichment analysis for the genes showing upregulated and downregulated A5SS events in human dCD8 T cells. (A-D) Venn diagram (A) and GO annotation (B-D) for the genes showing upregulated (FDR < 0.05 with $\Delta\Psi > 0.05$) and downregulated (FDR < 0.05 with $\Delta\Psi < -0.05$) A5SS events in human dCD8 T cells with respect to autologous pCD8 T cells. GO, Gene Ontology; A5SS, alternative 5' splice site; FDR, false discovery rate.

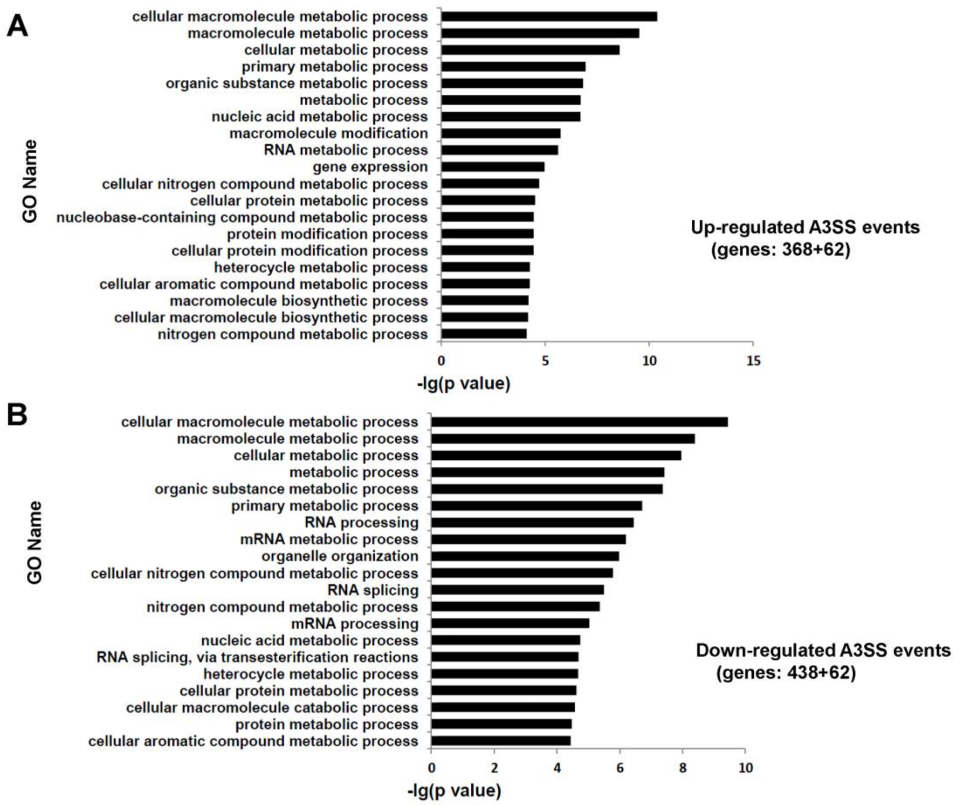

Figure S17. GO annotation for the genes undergoing upregulated or downregulated A3SS events in human dCD8 T cells. (A-B) The top 20 GO terms enriched for the genes undergoing upregulated (FDR < 0.05 with $\Delta\Psi > 0.05$ between samples; A) and downregulated (FDR < 0.05 with $\Delta\Psi < -0.05$ between samples; B) A3SS events in human dCD8 T cells with respect to pCD8 T cells. GO, Gene Ontology; A3SS, alternative 3' splice site; FDR, false discovery rate.

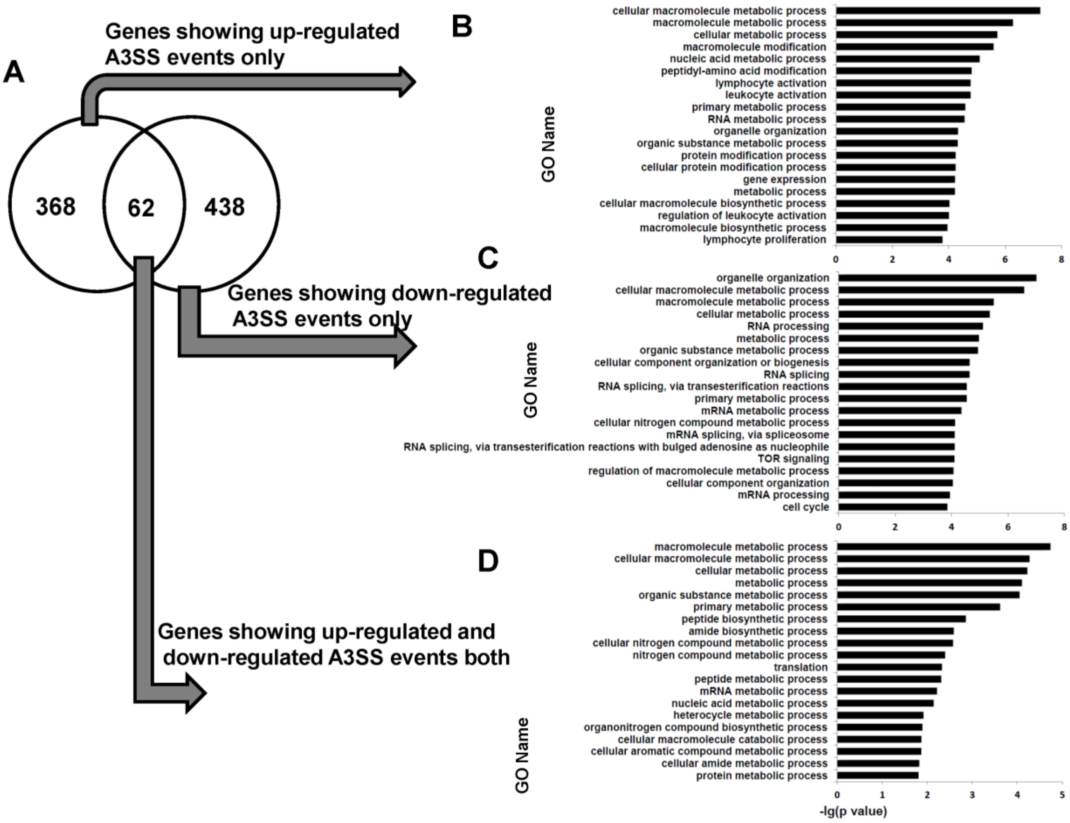

Figure S18. Venn diagram and functional enrichment analysis for the genes showing upregulated and downregulated A3SS events in human dCD8 T cells. (A-D) Venn diagram (A) and GO annotation (B-D) for the genes showing upregulated (FDR < 0.05 with $\Delta\Psi > 0.05$) and downregulated (FDR < 0.05 with $\Delta\Psi < -0.05$) A3SS events in human dCD8 T cells with respect to autologous pCD8 T cells. GO, Gene Ontology; A3SS, alternative 3' splice site; FDR, false discovery rate.

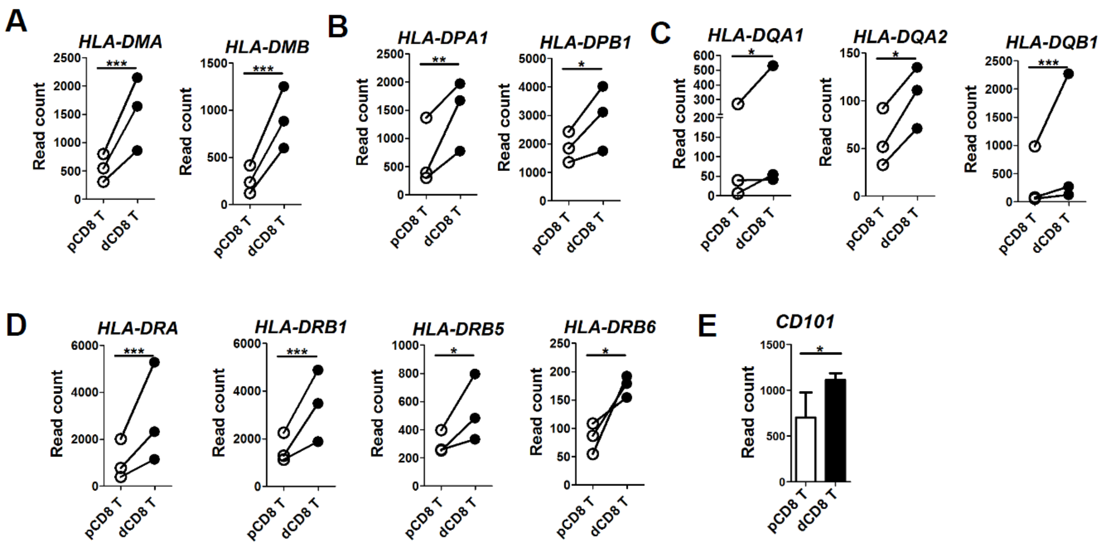

Figure S19. Comparison of the gene expression of MHC-II molecules (A-D) and *CD101* (E) between human paired pCD8 and dCD8 T cells. Gene expression quantification is measured as the read count. Each symbol reflects a sample and each line reflects the samples from the same individual (n = 3 per group). Differential expression analysis was performed using DESeq2 algorithm (paired test). pCD8 T, peripheral blood CD8^{+} T; dCD8 T, decidual CD8^{+} T.

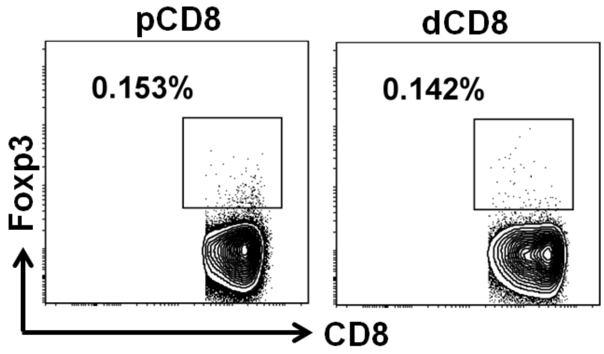

Figure S20. Foxp3^{+} cells are extremely few in both human pCD8 and dCD8 T cells. Representative flow cytometric plots illustrating the expression of Foxp3 in paired pCD8 and dCD8 T cells. Similar results were obtained from four individuals at the first trimester of normal pregnancy. pCD8 T, peripheral blood CD8^{+} T; dCD8 T, decidual CD8^{+} T.
